# Communication competences of multiple sclerosis neurologists during advance care planning conversations: A multi-observer study

**DOI:** 10.1371/journal.pone.0336183

**Published:** 2026-03-12

**Authors:** Andrea Giordano, Ludovica De Panfilis, Roberta Martina Zagarella, Giulia Di Domenico, Mariangela Farinotti, Alberto Gajofatto, Maria Grazia Grasso, Alessandra Lugaresi, Sara Montepietra, Francesco Patti, Claudio Solaro, Riccardo Orlandi, Giorgia Presicce, Simona Toscano, Simone Veronese, Lidia Del Piccolo, Alessandra Solari, on behalf of the ConCure-SM project

**Affiliations:** 1 Unit of Neuroepidemiology, Fondazione IRCCS Istituto Neurologico Carlo Besta, Milan, Italy; 2 Neurology, Public Health and Disability Unit, Fondazione IRCCS Istituto Neurologico Carlo Besta, Milan, Italy; 3 Department of Medical and Surgical Sciences, University of Bologna, Bologna, Italy; 4 Azienda Ospedaliero-Universitaria IRCCS, Bologna, Italy; 5 National Research Council (CNR), Interdepartmental Center for Research Ethics and Integrity (CID Ethics), Rome, Italy; 6 Department of Neuroscience, Biomedicine and Movement Sciences, University of Verona, Verona, Italy; 7 Unit of Neurology, Borgo Roma Hospital, Azienda Ospedaliera Universitaria Integrata Verona, Verona, Italy; 8 Multiple Sclerosis Unit, IRCCS S. Lucia Foundation, Rome, Italy; 9 IRCCS Istituto delle Scienze Neurologiche di Bologna, Bologna, Italy; 10 Dipartimento di Scienze Biomediche e Neuromotorie, Università di Bologna, Bologna, Italy; 11 Multiple Sclerosis Center, Azienda USL-IRCCS di Reggio Emilia, Reggio Emilia, Italy; 12 University Hospital Policlinico Vittorio Emanuele, Catania, Italy; 13 Neurology Unit, Galliera Hospital, Genoa, Italy; 14 Fondazione FARO ETS, Turin, Italy; 15 Section of Clinical Psychology, Department of Neurosciences, Biomedicine and Movement Sciences, University of Verona, Verona, Italy; Murcia University, Spain, SPAIN

## Abstract

**Background:**

The ConCure-SM intervention [ISRCTN48527663] consists of an advance care planning (ACP) training program for neurologists/other professionals caring for people with progressive multiple sclerosis (PwPMS). We assessed the communication competences of ACP-trained neurologists who participated in the trial.

**Methods:**

Eighteen ACP conversations were audio-recorded. After each conversation, participants (PwPMS, significant others [SOs], and neurologists) rated the neurologist’s communication skills using dedicated versions of the Quality of Communication questionnaire (QOC). Independent observers assessed the conversations using the Observing Patient Involvement in Shared Decision Making (SDM; OPTION), and the Verona Coding Definitions of Emotional Sequences (VR-CoDES).

**Results:**

Mean duration of the ACP conversations was 62.7 minutes. Neurologists (5/7 women) were 30–62-year-old. PwPMS mean age was 61.8 years, 39% were women. Mean QOC scores (distinguished in general communication and end-of-life communication; 0–100 scale) were 92.4, 90.1 respectively, according to PwPMS; 95.2, 94.3 according to SOs. Mean neurologists’ self-reported QOC score (0–100 scale) was 69.5. Mean OPTION score (0–100 scale) was 48.4. PwPMS expressed a median of 8.5 cues (hints to emotion) and 3.0 concerns (explicit expressions of emotion) per conversation. Neurologists provided space to 59% of cues and 73% of concerns. A quarter of cues/concerns contained figurative expressions or metaphors to express concerns or as signals of emotional distress. Moreover, 12% of expressions were coded as positive emotional statements (i.e., expressions indicating hope, confidence, and positive wishes).

**Conclusions:**

Neurologists’ communication skills during ACP conversations were rated as high using the QOC by both PwPMS and SOs, while neurologists rated their own skills more critically. Neurologists’ SDM competences were moderate, but higher compared to two published studies on MS consultations. Qualitative and quantitative analysis of ACP conversation transcripts indicate that ACP can evoke many emotions. In most instances neurologists provided space for the PwPMS to elaborate on affective expressions.

**Trial registration:**

ISRCTN48527663.

## Introduction

People with a progressive form of Multiple Sclerosis (MS) may live for many years while experiencing a wide range of symptoms, impairments (including cognitive deficits), and comorbidities [1]. For these reasons, they may benefit from Advance Care Planning (ACP), not only because it can lead to the creation of a legally binding document, but also because it represents an ongoing communicative process focused on identifying personal values and meanings to support informed and conscious health decisions.

The emphasis on patients’ values, preferences, and wishes is a core feature of ACP, as well as of shared decision making (SDM). SDM is a collaborative approach by which, in partnership with their HP, patients are encouraged to think about the available care options and the likely benefits and harms of each, to communicate their preferences, and help select the best course of action that fits these [2]. SDM mostly focuses on short-to-mid-term goals of care, while ACP on long-term goals of care, in the event of an impairment of decisional capacity. From MS diagnosis many key decisions are to be made by persons with MS. Most of such decisions are preference-sensitive, which makes SDM particularly important. Some MS clinical guidelines recommend incorporating patient preferences into disease-modifying treatment decisions [3]. However, evidence of implementation of SDM in routine MS care is limited [4–6].

SDM has been recently proposed as a ‘method of care’ for HPs and patients in any disease phase or care setting, including conversations to elicit patient-centered goals for end-of-life care in the acute setting [7,8].

Concerning ACP, discussing life goals and preferences, quality of life, dying, and self-determination can enhance satisfaction with communication between HPs, patients, and their families [9,10]. According to Pedrosa Carrasco et al. (2021) [10], ACP can improve patients’ awareness of their priorities and preferences, as well as their sense of engagement in the illness journey. Moreover, ACP can strengthen family relationships and foster trust and understanding between patients and HPs [11]. Also, emotions play a central role in the ACP process. There are few studies analyzing the impact of ACP on the care of people with MS, and even fewer that highlight the emotions that arise during ACP conversations between patients and their HPs. Among these studies, Tay et al. (2021) observed that during ACP conversations emotional expression with positive and negative valence emerged [12]. These emotions arose because conversations focused on positive and negative decisional, relational, and existential topics. Moreover, according to the literature the patients’ perception of ACP involvement seems to be associated with positive emotional functioning [13].

This manuscript reports the analysis of the first ACP conversation between people with progressive MS (PwPMS) and their neurologists in the ConCure-SM pilot trial (ISRCTN48527663) [14]. Briefly, ConCure-SM was a single-arm pilot and feasibility study on a structured ACP intervention, consisting of: I) a training program on ACP for neurologists and other MS HPs (S1 Appendix); II) a booklet to be used during the ACP conversation, which was co-produced with PwPMS and MS HPs [15]. The intervention supported neurologists in guiding PwPMS in their ACP. However, we found a worsening of PwPMS anxiety at both one and six-month follow-ups [14]. For this reason, we decided to add the analysis of the emotional contents of the conversations to the planned analysis of information exchange.

## Materials and methods

We analyzed the first ACP conversation between PwPMS and their (trained) neurologist. Another HP could participate in the conversation, as well as a PwPMS significant other (SO). Each conversation was audio-recorded and transcribed verbatim. After the conversation, PwPMS completed the Quality of Communication Questionnaire (QOC) [16], SOs completed the SO version (QOC-SO), and neurologists the physician version (QOC-DOC; last two items of the QOC).

### Ethics

The study protocol was approved by the ethics committees of the coordinating center (Fondazione IRCCS Istituto Neurologico Carlo Besta; 14.04.2021, internal ref: 83/2021), and all the six enrolling centers: Verona (27.09.2021, internal ref: 55917); Moncrivello (15.07.2021, internal ref: 15210); Reggio Emilia (23.06.2021, internal ref: 80829); Bologna (14.09.2021, internal ref: 90076); Rome (22.06.2021, internal ref: 921); Catania (19.10.2021, internal ref: 47839). The study was carried out in accordance with the Good Clinical Practice principles and the Declaration of Helsinki recommendations. All participants (PwPMS, SOs, neurologists, and other HPs) gave written informed consent. The trial dataset was accessed by the study principal investigators and the data management/analysis team.

### Instruments

We assessed the quality of the first ACP conversation considering three perspectives: the PwPMS (and, when present, the SO), the neurologist, and properly trained independent observers. Third observers evaluated SDM, using the OPTION scale [17] and emotional communication, using the Verona Coding Definitions of Emotional Sequences (VR-CoDES) [18,19]. VR-CoDES assessment was not part of the original study protocol.

### The QOC

Developed from qualitative studies with patients, families, and clinicians, the QOC consists of 19 items measuring general communication (nine items) and communication about EOL care (eight items), each rated on a scale from 0 (‘the very worst I can imagine’/‘not at all’) to 10 (‘the very best I can imagine’/‘extremely’), or identified as something the clinician did not do. The 0/10 ratings are recoded to 1/11, with 0 imputed for ‘did not do’ (http://depts.washington.edu/eolcare/products/instruments/). We translated and culturally adapted the QOC into Italian, and produced a SO version (QOC-SO) and a physician version (QOC-DOC) which includes two questions – (1) ‘How comfortable do you feel when talking about dying?’ and (2) ‘Overall, how would you rate your communication with this patient during the ACP conversation?’ – combined in a total score (0–100) [20].

### The OPTION

The OPTION (https://www.glynelwyn.com/observer-option-12-2005-2013.html) is an observer-based scale that evaluates the behavior of the HP in terms of patient involvement in decision-making [16]. It consists of 12 items, each rated on a five-point Likert scale ranging from 0 (behavior not observed) to 4 (behavior observed to high standard). A total score (range 0–48) is obtained by adding the scores of each item. The scale is available in Italian [21], and has been used in the context of MS consultations [22].

### The VR-CoDES

The VR-CoDES is an observer-based coding system that aims to describe, in terms of sequence, how emotions emerge and are dealt with by the HP. It categorizes patient expressions of emotion as ‘concerns’ (clear, unambiguous verbalizations of unpleasant emotions), and ‘cues’ (verbal or nonverbal hints of unpleasant emotions). Cues are further divided into seven sub-categories (S1 Table). HP responses to cues and concerns are classified according to two major dimensions: explicitness (if the response mentions either the content/topic or the emotion in the cue or concern or both) and space provision for further disclosure [19]. The VR-CoDES was developed in Italian and has been used in the context of MS consultations [23].

### Procedure

We conducted a total of 19 first ACP conversations. One PwPMS refused the recording, thus 18 conversations were audio-recorded and transcribed verbatim. Before conversation, PwPMS completed the Hospital Anxiety and Depression Scale (HADS) [24,25].

Two researchers (AGi, AS) previously trained in the use of OPTION scale independently coded each transcript/recording, with differences resolved by discussion.

Three researchers (LuDP, RMZ, GDD) received the one-day VR-CoDES training by the author of the coding system (LiDP), and coded jointly three transcripts/recordings. The remaining 15 transcripts/recordings were coded independently by two researchers, and a third researcher was involved in case of coding discrepancies. All codes were then discussed with LiDP.

### Analyses

#### Quantitative analysis.

Categorical variables were summarized as counts and percentages and compared using the chi-squared test or Fisher’s exact test, as appropriate. Continuous variables were summarized as means and standard deviations (SDs), or medians and interquartile ranges (IQRs); they were compared using Kruskal-Wallis or Wilcoxon ranksum test. Inter-rater reliability was assessed with the intraclass correlation coefficient (ICC) with 95% confidence intervals (CI) [26]. Statistical analyses were performed with Stata 16 (Stata, College Station, Texas, USA).

#### Qualitative analysis.

We followed the thematic analysis by Braun and Clarke (2019) [27] to analyze cues and concerns emerging from the VR-CoDES analysis. We used the line-by-line coding, prioritizing participants’ words to maintain authenticity and ensure the model’s originality. Three researchers (RMZ, GDD and LuDP) independently analyzed cues and concerns by reading the transcriptions, extrapolating the sub-themes that emerged and grouping and/or dividing them into categories of content. An iterative process was used to verify the consistency of sub-themes and themes with the transcript, identifying significant sentences that condensed and represented the meaning of the sub-themes and the identified themes.

Discrepancies between the researchers’ categorizations were resolved through discussion, leading to a final, agreed-upon categorization.

Adherence to the pertinent domain of the Consolidated Criteria for Reporting Qualitative Research (COREQ) checklist [28] (Domain 3: analysis and findings) is documented in S2 Table.

## Results

Mean duration of the ACP conversations was 62.7 minutes (SD 18.3). Five conversations had two participants (the PwPMS and the neurologist), eight involved an additional participant (four the PwPMS SO, and four another HP), and five conversations involved two additional participants (the PwPMS SO and another HP). The general and clinical characteristics of the PwPMS and their SOs are reported in Table 1. PwPMS mean age was 61.8 years (range 50–77); 7 (39%) were women. Eleven (61%) had secondary progressive MS; median Expanded Disability Status Scale (EDSS) score was 8.0 (IQR 6.5–8.0). SOs were 57.1 (range 34–74) years old; 4/9 (44%) were women; six were the spouse, one the son, and two the brother of the PwPMS. The neurologists (5/7 women, 71%) were 30–62-year old and had 5–30 years of experience in MS care.

**Table 1:** Characteristics of the people with progressive multiple sclerosis (PwPMS) and their significant others.

Characteristic		PwPMS (n = 18)	Significant others (n = 9)
		No (%)	
Women		7 (39)	4 (44)
Age (years) – Mean, SD		61.8 (7.8)	57.1 (11.5)
Education:	Primary (5–8 years)	2 (11)	–
	Secondary (12–13 years)	13 (72)	6 (67)
College/University (14 + years)		3 (17)	3 (33)
Occupation: Retired (disability)		11 (61)	–
	Employed	3 (17)	5 (56)
	Retired (age)	3 (17)	2 (22)
	Housewife	1 (5)	2 (22)
Relation:	Spouse/partner		6 (67)
	Brother		2 (22)
	Son		1 (11)
Age at MS diagnosis (years) – Mean, SD		40.3 (14.4)	
MS type:	Primary progressive	7 (39)	
	Secondary progressive	11 (61)	
EDSS – Median (IQR)		8.0 (6.5-8.0)	
Barthel Index – Median (IQR)		28.5 (13.0–64.0)	
HADS Anxiety – Median (IQR)		4.0 (2.0–5.0)	
HADS Depression – Median (IQR)		4.5 (1.0–8.0)	

EDSS, Expanded Disability Status Scale; HADS, Hospital Anxiety and Depression Scale; IQR, interquartile range; MS, multiple sclerosis; SD, standard deviation.

### Quality of communication questionnaire (QOC)

PwPMS mean QOC subscale scores (0–100) were 92.4 (SD 9.6) for ‘general communication’ and 90.1 (SD 10.2) for ‘communication about EOL care’. SOs’ mean QOC-SO scores were 95.2 (SD 6.4) for ‘general communication’ and 94.3 (SD 7.0) for ‘communication about EOL care’. Finally, neurologist mean QOC-Doc score was 69.5 (SD 16.0).

### Shared decision making

Inter-rater reliability for the OPTION total score was good (ICC 0.80, 95% CI 0.54–0.92). Disagreement exceeded 3 points in four instances. At the item level, disagreement exceeded one point in three instances: one for “offering opportunities to ask questions” (item 9) and two for “eliciting preferred involvement in decision-making” (item 10). After discussing discordant items, the resulting mean OPTION total score was 48.4 (SD 7.5; median 46.9, IQR 43.8–54.2), corresponding to a moderate degree of patient involvement. The most frequently observed behaviors were: “drawing attention to an identified problem requiring a decision” (item 1) and “exploring the patient’s concerns” (item 7). By contrast, in no case did neurologists elicit the patient’s preferred approach to receiving information (item 3); this behavior was not observed as the ACP interview was in fact structured, with the use of the booklet (Table 2). A key step in the ACP dialogue is the (continuous) clarification and understanding of the patient’s values, to help the delivery of care that aligns with those goals and values [29–31]. As this behavior is not assessed in the OPTION scale, we have added the item “exploring patient’s values”, which was rated using the same five-point scale, and obtained a median score of 50 (IQR 50–75).

**Table 2:** Distribution of the Observing Patient Involvement in Shared Decision Making (OPTION) item and total scores.

Item no.	Behavior description	Median (IQR)
1	Drawing attention to an identified problem as one that requires a decision-making process	75 (50–75)
2	Stating that there is more than one way to deal with the identified problem (equipoise)	50 (50–75)
3	Assessing the patient’s preferred approach to receiving information to assist decision making (e.g., discussion, reading printed material, using videotape or other media)	0 (0–0)
4	Listing options, which can include the choice of “no action”	50 (50–75)
5	Explaining the pros and cons of options to the patient	50 (25–50)
6	Exploring the patient’s expectations (or ideas) about how the problem(s) is to be managed	50 (50–50)
7	Exploring the patient’s concerns (fears) about how the problem(s) is to be managed	62.5 (50–75)
8	Checking that the patient has understood the information	50 (50–50)
9	Offering the patient explicit opportunities to ask questions during the decision-making process	50 (25–75)
10	Eliciting the patient’s preferred level of involvement in decision making	50 (50–50)
11	Indicating the need for a decision making (or deferring) stage	50 (50–50)
12	Indicating the need to review the decision (or deferment)	50 (50–50)

IQR, interquartile range.

### Emotional communication

According to the VR-CoDES analysis, PwPMS expressed 179 cues and 63 concerns, while neurologists provided space for 59% of cues and 73% of concerns (Table 3). Most cues were of B (37%) and D (14%) type (S1 Table). Expressions indicating hope, confidence, and positive wishes were coded as positive emotional statements (PESs), which accounted for 12% of the instances.

**Table 3:** Distribution of concerns and cues (cue types detailed in S1 Table) according to whether spontaneous or neurologist-elicited and according to the neurologist’s immediate response (reduce vs. provide space).

	PwPMS expression			Neurologist’s response	
	No (column %)	Spontaneous	Neurologist-elicited	Provide space	Reduce space
		No (row %)		No (row %)	
Concern	61 (22.3)	11 (18.0)	50 (82.0)	44 (72.1)	17 (27.9)
Cue A	15 (5.5)	8 (53.3)	7 (46.7)	10 (66.7)	5 (33.3)
Cue B	101 (37.0)	50 (49.5)	51 (50.5)	55 (54.5)	46 (45.5)
Cue C	4 (1.5)	1 (25.0)	3 (75.0)	3 (75.0)	1 (25.0)
Cue D	37 (13.6)	18 (48.6)	19 (51.4)	27 (73.0)	10 (27.0)
Cue E	11 (4.0)	11 (100)	0	4 (36.4)	7 (63.6)
Cue F	2 (0.7)	0	2 (100)	2 (100)	0
Cue G	10 (3.7)	10 (100)	0	6 (60.0)	4 (40.0)
PES	32 (11.7)	15 (46.9)	17 (53.1)	22 (68.7)	10 (31.3)
Totals	273 (100)	124 (45.4)	149 (54.6)	173 (63.4)	100 (36.6)

PwPMS, people with progressive multiple sclerosis; PES, positive emotional statement.

A quarter of cues/concerns (61/242) contained figurative expressions: most of them were in type B cues, but they were also found in type A, D, E, and G cues, in concerns, and PES (Table 3). 34/61 (55.7%) of neurologists’ responses were coded as explicit; 41/61 (67.2%) provided space for the PwPMS to stay on the emotional topic or delve deeper into the cue/concern. The most frequently coded types of responses were: “acknowledges content” (e.g., PwPMS: “I’m the type of person who wants to know who I have to fight-with”. Neurologist: “Clearly, it’s not a process that started yesterday […], living with the disease is something that is well established in your life…”), “ignores” (PwPMS: “Since I cannot go out, sometimes my daughter has to take over […]. Because I was very active …”. Neurologist: “Yes. However, other issues, for example, that may concern others?”), “acknowledges affective aspect” (e.g., PwPMS: “the disease really cut my legs off”. Neurologist: “This vision of life is comprehensible, there’s no doubt about it”), and “brief facilitation, encouragement (e.g., Neurologist: “Yes”; “Hmm”).

### Use of figurative language

The recurring metaphor of *‘feeling like a burden’* encapsulates the physical and emotional burden imposed by the illness, reflecting the loss of independence and the associated guilt, particularly the regret of being a burden on loved ones. Furthermore, PwPMS often used the expression *‘being in a fight’* to convey the constant battle with their illness.

The expression *‘being a puppet’* vividly illustrates the loss of cognitive control, portraying the PwPMS as manipulated by external forces, thus highlighting the loss of autonomy and the feeling of being at the mercy of the disease. The expression *‘the disease is galloping’* conveys the rapid and relentless progression of the condition, suggesting an uncontrollable and overwhelming force. The metaphor *‘having no anchors’* represents the absence of stability and reference points in the PwPMS life, leading to a sense of being adrift. ‘*It looks grim’* poignantly expresses despair and hopelessness, indicating an inability to envision a positive future. Other significant metaphors include *‘floating’* to describe the need to learn to survive day by day and to adapt to circumstance; *‘I have seen pieces of my body fall away’* to depict the progressive loss of physical functioning; and *‘having the legs taken away by the disease’* to illustrate its impact on their lives. The metaphor *‘body as a shell’* portrays the feeling of being an empty vessel, devoid of vitality. The metaphors *‘running on empty’* and the *‘curve of suffering’* refer to the exhaustion caused by the illness. *‘Being with my four-way indicators on’* describes a minimal vital state; and *‘holding my son back with the handbrake on’* metaphorically describes the act of limiting one’s SO due to the constraints imposed by the illness.

Finally, ACP is described as *‘closing a door’*, which refers to the relief of having addressed what your (end of) life may hold given the illness. Similarly, *‘ACP as knowing you have defused a problem’* and *‘ACP as removing a variable’* indicate the relief of having managed a specific aspect of the illness.

### Thematic analysis

We identified four major themes that emerged from the emotional communication codes: the impact of the illness on PwPMS and SOs’ lives, the possible loss of personal autonomy, the caring approach of the physician, and feelings related to discussing EOL. Selected quotes supporting the four themes are reported in Table 4.

**Table 4:** Selected quotes supporting the four themes.

Theme	PwPMS code	Quote
Impact of the illness on personal and SOs’ lives	P115	It looks grim. Many times, I think the only path I can take is to die, so I don’t have to think anymore. And most of all, it really bothers me to make the people who care about me suffer.
	P209	So, considering how fast the disease is galloping, [what worries me most] is the worsening. Especially losing my cognitive abilities; because now my legs are gone, they’re basically just there for show. My left arm is also kind of just there – not fully, but almost. And what I have left that’s still functioning is my head and the right side of my body. And if I lose those too… What’s left of me? How can I put it? I mean, for me, flesh and bones – they’re just a shell…
	P605	Maybe what worries me most is not always being able to rely on him. I mean, right now it’s easy to rely on him. But as time goes by, it’ll get harder. Harder for me, because obviously I’ll get worse. Harder for him, because he’s getting older too. And I’m not easy to handle, since I’m physically quite large.
Possible loss of personal autonomy	P126	My worry is that I don’t want to feel physical pain. I’ve been worried because I’ve seen parts of my body go. One leg broken, then the other; a broken hand […]. And now I’m afraid of losing the use of this other hand, of losing mental clarity, and the ability to speak.
	P606	I’m not autonomous even now – not at all, not even a little. I can’t even shoo a fly away from my face. Nothing. I need someone to blow my nose for me. To feed me. […] So I’m at a bad point. But all things considered, as long as your mind still works… But if something else goes, then what do you do? For example, if you can’t talk anymore, or even eat… what meaning do you have? You don’t have any meaning, not for yourself […] and not for others who are suffering. I have only one son, and I keep holding him back with the handbrake on…
Caring approach of the physician	P502	You can keep going. Thanks to our strength, but above all thanks to the doctors who help us, because they study a lot, they’re helping us.
	P507	I’ve always trusted medicine, and therefore the doctors, when it comes to this disease. […] With experience – over time – and by gathering information on my own, I realized that medicine didn’t really inform me much, not until then. Meeting you, I finally received more attention in terms of having the illness explained to me. OK? Maybe until that point I hadn’t really needed that kind of attention. Probably, as the disease worsened, there was a kind of synergy, let’s say, in understanding each other, in stimulating each other – to explain more, to ask one more question, to give one more answer. And so, that’s definitely a good thing. Before, that didn’t happen, and I kind of regret it. I mean, from that point of view, I felt less supported. I was monitored, for sure – but not really advised, not encouraged to explore new treatments or medications, or whether they were worth taking or not.
EOL discussion	P302	This tool – for me – it’s a moment, not exactly pleasant, but… useful. For me, it’s positive. […] Just knowing that this tool exists, and that I can close that door – at least that part of the illness – overall it gives me comfort. That’s something positive.
	P402	These are… really difficult questions. But you have to ask them anyway. […] I’m glad I did it [the ACP], though I didn’t expect some of the questions…

ACP, advance care planning; EOL, end of life; PwPMS, person with progressive multiple sclerosis; SO, significant other.

PwPMS expressed emotions related to the current impact of MS on their lives in terms of the need to adapt to and try to overcome the barriers imposed by the disease: they expressed both negative emotions, namely frustration about feeling overwhelmed by their MS, and positive emotions about developing new awareness on their personal resources. PwPMS also expressed the fear of being a burden to others or of a negative impact of their MS on relationships and friendships. The ACP conversation produced a sense of relief and self-confidence as it meant making decisions for themselves, without leaving beloved ones the burden of making choices.

The ACP conversation provided an opportunity to discuss the possible evolution of their disease and the related fear of losing autonomy. The concept of personal autonomy was manifested in two ways: the fear of becoming physically dependent on others in any daily activities, and concerns about losing mental capacity.

Emotions related to the caring approach of the physician were reported as anger or feelings of abandonment by PwPMS who, during the illness pathway, had to make health decisions alone, while other PwPMS expressed gratitude to be cured by a physician with “a good soul” and able to have a deep communication with them.

Finally, some PwPMS were comforted and calm during the structured conversation dedicated to EOL issues. In contrast, others expressed negative emotions such as anxiety. Some PwPMS tried to avoid these thoughts and found the scenarios related to EOL choices too challenging.

## Discussion

Findings of our pilot and feasibility trial on a training program on ACP for neurologists and other MS HPs have been recently published [14]. ACP training programs for HPs have been developed and tested in various disciplines, including oncology [32–34], and internal medicine [35] where recent evidence shows that clinician training had a positive effect on ACP uptake and documentation. Further, preliminary evidence in ALS [36] and Parkinson’s disease [37,38] suggests that integrating palliative care with ACP is feasible and valuable for patients and clinicians.

Here we report the communication competences of neurologists who participated in our pilot trial. Eighteen first ACP conversations of PwPMS with seven neurologists were transcribed and analysed. Physicians well varied in age and years of MS experience; none of them had any previous experience of ACP.

We assessed the perspectives of the diverse participants (PwPMS, SOs, and neurologists) using corresponding versions of the QOC scale [20]. Moreover, these competences were assessed by independent observers considering two pivotal elements of medical communication: information exchange/SDM (using the OPTION) and emotional communication (using the VR-CoDES).

Mean QOC scores of PwPMS and SOs were high (around 90) for both ‘general communication’ and ‘communication about EOL care’ subscales, while neurologists were more self-critical, with mean values below 70. Although PwPMS and SOs completed the QOC using a dedicated platform (or by an interviewer) to guarantee confidentiality it is possible that the high scores obtained were affected by acquiescence bias. Concerning the lower QOC-Doc scores, this can be explained by the fact that ACP was a new practice for all the participating centers, and neurologists were at their first ACP experience after the training, out of the comfort zone of a typical MS consultation.

The mean OPTION total score was 48.4, corresponding to a moderate degree of SDM competences of our neurologists. This value was higher compared to the two studies that, to our knowledge, have used the OPTION in the context of consultations at MS centers in Italy [22] and Germany [39], both with mean scores around 30. Recently, a large cluster randomized controlled trial involving 65 Belgian nursing home wards applied the OPTION to audio recordings of ACP conversations between residents, families and staff [40]. Wards assigned to SDM training showed an increase in mean OPTION score from 27 to 54 (p < 0.001) while time spent on the conversations did not increase. This improvement persisted six months after training (mean OPTION score 56; p < 0.001) [40].

The VR-CoDES revealed two main findings. First, PwPMS expressed a median of 8.5 cues and 3 concerns per conversation, and these expressions were higher compared to a study of first consultations at Italian MS centers (median 4 cues and 1 concern per consultation) [23]. This indicates that the ACP conversation can elicit a huge number of patient emotions, which is consistent with the increase in PwPMS anxiety symptoms as assessed using the Hospital Anxiety and Depression Scale (p = 0.02), and with the qualitative study nested in the trial, revealing the ‘emotional cost’ of ACP for both PwPMS and their SOs [14]. Second, neurologists provided space to 59% of cues and 73% of concerns. This differed from Del Piccolo et al. (2015) study, where corresponding figures were 42% for cues and 24% for concerns, and common neurologists’ reactions included changing the subject, taking no notice, and giving medical advice [23]. This suggests that the ConCure-SM training program helped neurologists to provide space to the many emotions that emerged and to show a meaningful engagement in the ACP conversation.

Emotions that emerged were not only related to the disease and treatment decisions, but also to broader aspects of patients’ lives, such as the loss of personal autonomy, the individual resources in managing the disease, and the overall impact of the disease on personal identity. This is particularly noteworthy, as it demonstrates that ACP is not merely a tool for obtaining consent for healthcare choices, but a process through which personal values and life perspectives are more deeply understood, articulated, made explicit and shared.

Interestingly, positive PwPMS emotions also emerged, including expressions of hope, confidence, and positive expectations, suggesting a potentially beneficial role of ACP.

The emotional burden of the ACP process emerged through the figurative language used during the conversations. These expressions helped PwPMS to share how they perceive themselves within the context of the disease. Our results are consistent with studies performed in other settings, showing the occurrence of both positive and negative emotional expressions during ACP discussions and more generally during EOL conversations [12,41]. Two studies also reported an increased patient trust in the care team and a stronger sense of closeness with the clinician because of ACP conversation [42,43].

Our study has some limitations. First, our sample was small with 18 ACP conversations included in the analysis, which may limit the generalizability of our findings to a broader population of neurologists or PwPMS. Moreover, the study was conducted in Italian MS centers, which may not account for cultural or contextual differences in other countries or healthcare settings.

In conclusion, our multi-perspective analysis of the first ACP conversations document good communication competences of MS neurologists, exhibited in a highly emotive context. Our findings support the value of training programs to improve the confidence of neurologists in ACP practice. Further research should explore strategies to implement this practice out of the setting of a pilot trial, in the busy environment of an MS center.

## Supporting information

S1 Appendix The ConCure-SM training. (PDF)

S1 Table The seven sub-categories of cues in the Verona Coding Definitions of Emotional Sequences (VR-CoDES). (DOCX)

S2 Table The Consolidated Criteria for Reporting Qualitative Research (COREQ) checklist. (DOCX)
