# Supplementary material for: Communication competences of multiple sclerosis neurologists during advance care planning conversations: A multi-observer study
Source: PLoS One. 2026 Mar 12;21(3):e0336183. doi: 10.1371/journal.pone.0336183 (PMC12981511; doi:10.1371/journal.pone.0336183)
Supplement: S1 Appendix — (PDF) [file pone.0336183.s001.pdf]

S1 APPENDIX

The ConCure-SM training

ConCure-SM*

Training dei Professionisti Sanitari

Milano, AmadeoLab

Agenda

Venerdì,15 ottobre

10:00-10:30	Ice breaking	
10:30-11:00	Pre-test	
11:00-11:30	SHARED DECISION-MAKING	K Mattarozzi, A Solari
11:30-11:50	CAPACITÀ COGNITIVA	E Pucci
11:50-12:50	PIANIFICAZIONE CONDIVISA DELLE CURE	M Cascioli, L De Panfilis, S Veronese
12:50-13:20	Discussione	
13:20 14:00-	Light lunch	
15:30 15:30-	GoWish & AUTO-CONSAPEVOLEZZA	L De Panfilis, M Rimondini
16:00 16:00-	Introduzione ai laboratori	S Veronese
17:00 17:00-	LABORATORIO 1 & LABORATORIO 2	
18:00	Discussione	

Sabato, 16 ottobre

8:30-9:30	LABORATORIO 3 & LABORATORIO 4
9:30-10:30	Discussione
10:30-11:15	LABORATORIO 5
11.15-12:00	Discussione
12:00-12:30	Post-Test e questionario ECM
12:30	Light lunch

Docenti

Dott. Marta Cascioli Infermiera Palliativista– *Azienda USL Umbria 2, Spoleto*

Dott. Ludovica De Panfilis Bioeticista –*Azienda USL-IRCCS di Reggio Emilia*

Prof. Katia Mattarozzi Psicologa –*Università di Bologna*

Dott. Eugenio Pucci MedicoNeurologo –*Ospedale di Fermo*

- Prof. Michela Rimondini Psicologa – *Università di Verona*
- Dott. Alessandra Solari Medico Neurologo – *Fondazione IRCCS Istituto Neurologico Carlo Besta, Milano*
- Dott. Simone Veronese Medico Palliativista – *Fondazione F.A.R.O. Onlus, Torino*

*BMJ Open. 2021;11(8):e052012

ConCure-SM

Training dei Professionisti Sanitari
Amadeo Lab, 15-16 ottobre 2021

SHARED DECISION-MAKING

Katia Mattarozzi e Alessandra Solari

SCLE ROSI MULT IPLA associazione italiana
un mondo libero dalla SM

Fondazione I.R.C.C.S. Istituto Neurologico Carlo Besta

Sistema Socio Sanitario Regione Lombardia

Decisioni sensibili alle preferenze:

- Evidenza incerta o assente a supporto della maggiore efficacia di un’opzione terapeutica su un’altra

- Ogni opzione terapeutica ha un diverso rapporto benefici/rischi

- I valori, l’esperienza, le conoscenze del paziente sono importanti per la decisione migliore possibile

Stiggelbout AM, Van der Weijden T, De Wit MP, Frosch D, Légaré F, Montori VM, Trevena L, Elwyn G. Shared decision making: really putting patients at the centre of healthcare. BMJ. 2012 Jan 27;344:e256. doi: 10.1136/bmj.e256. PMID: 22286508.

Il paziente

- Esperienza di malattia
- Condizioni di vita
- Attitudine al rischio
- Valori
- Preferenze

Il clinico

- Diagnosi
- Eziologia
- Prognosi
- Opzioni terapeutiche
- Probabilità degli esiti

La CONOSCENZA del paziente ...

- Soggettiva
- Ipotetica
- Emotiva

Basata sulla PERCEZIONE

Diversa valutazione di:

- rischio
-probabilità di occorrenza
- esiti
- controllo

La CONOSCENZA del clinico ...

- Oggettiva
- Analitica
- Razionale

Basata sul RAGIONAMENTO

La SM è una malattia cronica, caratterizzata da una riduzione variabile dell’aspettativa di vita (tra 7 e 14 anni) rispetto alla popolazione generale, e da un decorso altrettanto variabile. Nella forma progressiva di malattia, i sintomi e le limitazioni

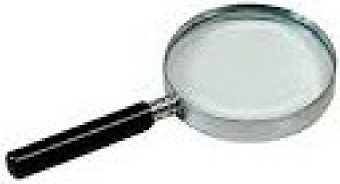

Mangset M, Berge E, Førde R, Nessa J, Wyller TB. "Two per cent isn't a lot, but when it comes to death it seems quite a lot anyway": patients' perception of risk and willingness to accept risks associated with thrombolytic drug treatment for acute stroke. J Med Ethics. 2009 Jan;35(1):42-6. doi: 10.1136/jme.2007.023192. PMID: 19103942.

Lloyd A J. The extent of patients' understanding of the risk of treatments. Quality in Health Care 2001;10(Suppl I):i14±i18

Hofmann A, Stellmann JP, Kasper J, Ufer F, Elias WG, Pauly I, Repenthin J, Rosenkranz T, Weber T, Köpke S, Heesen C; MS Network Hamburg. Long-term treatment risks in multiple sclerosis: risk knowledge and risk perception in a large cohort of mitoxantrone-treated patients. Mult Scler. 2013 Jun;19(7):920-5. doi: 10.1177/1352458512461967. Epub 2012 Oct 4. PMID: 23037548.

‘Buone pratiche’ di attuazione della SDM

1. Formazione dei clinici

2. Formazionedeipazienti

- dare consapevolezza dell’equipoise dare
- consapevolezza dell’importanza delle preferenze del paziente
- accrescere la partnership accrescere l’auto-efficacia del paziente
- esplorare le preferenze del paziente sul ruolo decisionale *

The Control Preference Scale

Solari A, Giordano A, Kasper J, Drulovic J, van Nunen A, Vahter L, Viala F, Pietrolongo E, Pugliatti M, Antozzi C, Radice D, Köpke S, Heesen C; AutoMS project. Role Preferences of People with Multiple Sclerosis: Image-Revised, Computerized Self-Administered Version of the Control Preference Scale. PLoS One. 2013 Jun 18;8(6):e66127. doi: 10.1371/journal.pone.0066127. PMID: 23823627; PMCID: PMC3688863.

CONSENSO INFORMATO: decisione in linea con la migliore evidenza disponibile

SCELTA INFORMATA (SDM): decisione in linea con la migliore evidenza disponibile, e con I valori e le preferenze del paziente (informato)

Persone diverse hanno atteggiamenti diversi nei confronti della propria salute, che le portano ad attribuire valori diversi ai comportamenti legati alla salute.
Pertanto, per alcune persone una scelta informata può essere la scelta di non intraprendere un comportamento o un trattamento, anche se consigliato dal proprio medico

Gli elementi del processo di SDM

1. Definire il ‘problema’
2. Asserire la sussistenza di equipoise
3. Illustrare le diverse opzioni
4. Informare su benefici e rischi di ciascuna opzione
5. Verificare la comprensione
6. Aprire a idee, preoccupazioni e attese d
7. Identificarele sue preferenze
8. Deliberare o deferire la decisione
9. Pianificare il follow-up

chrome-extension://efaidnbmnnnibpcajpcglclefindmkaj/https://www.gmc-uk.org/cdn/documents/good-medical-practice-2024---english_pdf-102607294.pdf

The OPTION Scale

Elwyn G, Hutchings H, Edwards A, Rapport F, Wensing M, Cheung WY, et al. The OPTION scale: measuring the extent clinicians involve patients in decision-making tasks. Health Expect. 2005;8(1):34-42.

https://www.glynelwyn.com/observer-option-12-2005-2013.html)

Pietrolongo E, Giordano A, Kleinefeld M, Confalonieri P, Lugaresi A, Tortorella C, et al. Decision-making in multiple sclerosis consultations in Italy: third observer and patient assessments. PLoS One. 2013;8(4):e60721. doi:10.1371/journal.pone.0060721.

Scaletta

- Disturbi cognitivi nella SM
- Principio bioetico di autonomia e capacità decisionale
- Le PCC e la «capacità»
- Standard per valutare le capacaità decisonali e strumenti
- Aggionmento delle PCC
- Barriere specifiche?
- Dimensione collegiale?

La disabilità nella sclerosi multipla

AISM che ha stimato in circa 6200 le persone con SM italiane viventi con *EDSS* > 8.5 (AISM 2017)

Almeno fino a qualche anno fa, ancora un tabù...

Westervelt HJ. Dementia in multiple sclerosis: why is it rarely discussed? Arch Clin Neuropsychol. 2015 Mar;30(2):174-7. doi: 10.1093/arclin/acu095. Epub 2015 Jan 24. PMID: 25618134.

Branco M, Ruano L, Portaccio E, Goretti B, Niccolai C, Patti F, Chisari C, Gallo P, Grossi P, Ghezzi A, Roscio M, Mattioli F, Bellomi F, Simone M, Viterbo RG, Amato MP. Aging with multiple sclerosis: prevalence and profile of cognitive impairment. Neurol Sci. 2019 Aug;40(8):1651-1657. doi: 10.1007/s10072-019-03875-7. Epub 2019 Apr 23. Erratum in: Neurol Sci. 2020 Jan;41(1):243. doi: 10.1007/s10072-019-04134-5. PMID: 31011932.

- Solo uno studio negli ultimi 10 aa che riporta prevalenza di demenza: 22% (criterio: un test di memoria + un altro dominio >2 SD sotto la media + compromissione dello stato occupazionale - Benedict and Bobholz, 2007)
- "*no informative prospective study permitting to establish the prevalence of dementia in MS*" (Defer & Branger 2015)

I disturbi cognitivi tendono ad aumentare nel decorso della SM
Uno studio longitudinale in 10 anni

Amato MP, Ponziani G, Pracucci G, Bracco L, Siracusa G, Amaducci L. Cognitive impairment in early-onset multiple sclerosis. Pattern, predictors, and impact on everyday life in a 4-year follow-up. Arch Neurol. 1995 Feb;52(2):168-72. doi: 10.1001/archneur.1995.00540260072019. PMID: 7848126.

Amato MP, Ponziani G, Siracusa G, Sorbi S. Cognitive dysfunction in early-onset multiple sclerosis: a reappraisal after 10 years. Arch Neurol. 2001 Oct;58(10):1602-6. doi: 10.1001/archneur.58.10.1602. PMID: 11594918.

La EDSS non è sensibile ai disturbi cognitivi

Saccà F, Costabile T, Carotenuto A, Lanzillo R, Moccia M, Pane C, Russo CV, Barbarulo AM, Casertano S, Rossi F, Signoriello E, Lus G, Brescia Morra V. The EDSS integration with the Brief International Cognitive Assessment for Multiple Sclerosis and orientation tests. Mult Scler. 2017 Aug;23(9):1289-1296. doi: 10.1177/1352458516677592. Epub 2016 Nov 3. PMID: 27811338.

Disturbi cognitivi come indicatore prognostico?

Moccia M, Lanzillo R, Palladino R, Chang KC, Costabile T, Russo C, De Rosa A, Carotenuto A, Saccà F, Maniscalco GT, Brescia Morra V. Cognitive impairment at diagnosis predicts 10-year multiple sclerosis progression. Mult Scler. 2016 Apr;22(5):659-67. doi: 10.1177/1352458515599075. Epub 2015 Sep 11. PMID: 26362896.

Pitteri M, Romualdi C, Magliozzi R, Monaco S, Calabrese M. Cognitive impairment predicts disability progression and cortical thinning in MS: An 8-year study. Mult Scler. 2017 May;23(6):848-854. doi: 10.1177/1352458516665496. Epub 2016 Aug 15. PMID: 27527906.

Confonditori... Nel nostro contesto altri ostacoli...

Cognitive impairment, fatigue, and depression in MS

Brochet et al., available at

Addressing Challenges in Unmet Needs

Independent medical education for global healthcare professionals to acquire new clinical skills, test their...

peervoice.com

Autonomia e capacità decisionale

Perché il malato partecipi alle scelte...
Perché l'operatore rispetti l'autonomia del malato...
Per condividere il progetto di cura...

Ci vengono in aiuto gli elementi del consenso:

- –Scelta volontaria
- –Informazione ("quanta?", "ben data", "ben compresa", etc)
- –Capacità decisionale

Beauchamp T, Childress J. *Principles of Biomedical Ethics.* 5th ed. New York, NY: Oxford University Press; 2001. Faden RR, Beauchamp TL. A History and Theory of Informed Consent. New York, Oxford University Press, 1986.

Si può fare una PCC con una persona che considero “incompetente”?

- Nell’art 5 relativo alla PCC non si definisce la “capacità” decisionale del pz ma si afferma “Per quanto riguarda gli aspetti non espressamente disciplinati dal presente articolo si applicano le disposizioni dell’articolo 4”.
- All’Art. 4 sulle DAT è citato: “Ogni persona maggiorenne e capace di intendere e di volere”

Questo lascia al medico un ampia responsabilità e discrezionalità nel decidere come e se impostare la relazione con il malato che è il fondamento (anche legale) delle PCC, quali temi e contesti decisionali possono essere espressione delle PCC, quali modalità e tempi…

Capacità: in senso «clinico» vs «legale»

- Capacità in senso «clinico»: il malato è in grado di decidere su una determinata scelta (indipendentemente dalla condivisione del medico e dall'indicazione clinica relativa a tale decisione)
- Solo i giudici hanno il diritto di dichiarare una persona «incapace» sul piano legale («**interdizione**» e «**inabilitazione**», indicando che tale persona necessita che altri decidano per lei - tutore legale o curatore)
- La capacità considerata in tali termini legali risulta di tipo categoriale, dicotomica
- In realtà concetto complesso e fluttuante, contesto-specifico
- Il medico dichiara «incapace» nella pratica clinica, quando ad esempio giudica un pz non i grado di fornire il consenso ad un trattamento e procede in urgenza
- In ogni caso il medico che ha in cura il malato è coinvolto.

continuum

CAPACITA' DECISIONALE

presente

Contesto

socio - culturale

assente

Contenuto e conseguenze della decisione

Variabile nel tempo

La demenza non comporta inevitabilmente la sua assenza

La regola generale vuole che la capacità di un malato sia presunta presente fino a prova contraria.

Cosa significa “Ogni persona maggiorenne e capace di intendere e di volere”

- Capacità di intendere: comprendere il valore e le conseguenze delle proprie azioni
- Capacità di volere: poter fare le proprie scelte in modo consapevole e responsabile

- Interdizione: non possibile pensare ad una PCC
- Inabilitazione
- Amministrazione di sostegno

} potenzialmente possiamo predisporre una PCC (laddove ci siano capacità residue che consentano di manifestare una volontà consapevole in ordine alle scelte)

- Tutto il resto: tocca al medico decidere…

Standard per valutare le capacità decisionali

APA:Guidelines for Assessing the Decision-Making Capacities of Potential Research Subjects With Cognitive Impairment.

Am. J Psychiatry 155:11, 1998

Appelbaum PS, Grisso T Assessing patients' capacities to consent to treatment. N Engl J Med 319:163–168, 1988

Capacità di :

- ***comunicare una decisione***
- ***comprendere l'informazione rilevante***
- ***apprezzare la situazione e le sue verosimili conseguenze***
- ***manipolare l'informazione razionalmente***

L’esigenza di formalismo

- Ci sono strumenti per documentare la capacità al consenso
- Ne abbiamo bisogno per le PCC?

MacCAT-T Grisso, T., & Appelbaum, P. S. (1998). MacArthur competence assessment tool for treatment (MacCAT-T). Sarasota, FL: Professional Resource.

Janofsky JS, McCarthy RJ, Folstein MF. The Hopkins Competency Assessment Test: a brief method for evaluating patients' capacity to give informed consent. Hosp Community Psychiatry. 1992 Feb;43(2):132-6. doi: 10.1176/ps.43.2.132. PMID: 1572608.

Fazel S, Hope T, Jacoby R. Assessment of competence to complete advance directives: validation of a patient centred approach. BMJ. 1999 Feb 20;318(7182):493-7. doi: 10.1136/bmj.318.7182.493. PMID: 10024254; PMCID: PMC27742.

Un esempio dal campo della malattia di Alzheimer: nel *MCI* si consiglia di discutere precocemente ACP…

Frederiksen, K. S., Nielsen, T. R., Winblad, B., Schmidt, R., Kramberger, M. G., Jones, R. W., Hort, J., Grimmer, T., Georges, J., Frölich, L., Engelborghs, S., Dubois, B., & Waldemar, G. (2021). European Academy of Neurology/European Alzheimer's Disease Consortium position statement on diagnostic disclosure, biomarker counseling, and management of patients with mild cognitive impairment. European journal of neurology, 28(7), 2147–2155. https://doi.org/10.1111/ene.14668

Il prima e il dopo….

▸ **La durata della validità e il rinnovo periodico**

Le risposte possono differire su questo punto, a seconda delle patologie. Il rinnovo periodico delle direttive e i limiti della loro validità consentono di avvicinarsi alla situazione reale. Comunque nel caso di malattie per le quali le capacità cognitive del paziente si deteriorano progressivamente in un lungo periodo, deve essere possibile riferirsi ai desideri espressi in anticipo prima che la condizione cognitiva del paziente sia compromessa al punto che divenga impossibile un valido rinnovo dei desideri. Quando è precisato un termine di validità, le regole dovrebbero stabilire ciò che deve essere fatto quando le direttive anticipate scadono, senza che sia possibile per il paziente rinnovare i propri desideri. Possono, in tali circostanze, essere totalmente ignorate? Esse continuano comunque ad offrire indicazioni sui desideri della persona. In ogni caso è ampiamente accettata la possibilità di revocare le direttive anticipate.

chrome-extension://efaidnbmnnnibpcajpcglclefindmkaj/https://rm.coe.int/168039e8c2

Io credo che si possa rispondere: (i) documentando una volontà da parte del pz di far valere le sue decisioni attuali per il futuro, riconoscendo al medico di fiducia la competenza di saper giudicare il momento in cui il rinnovo delle PCC perde senso a causa di una compromissione irreversibile delle capacità decisionali; oppure (ii) nominando un amministratore di sostegno.

D’altra parte l’aggiornamento della PCC è importante…

- Per favorire l’”attualità” della PCC.
- In effetti il pz ha sempre più consapevolezza “vissuta” dei sintomi che lo affliggono, e dei bisogni correlati, conseguenti all’evoluzione della malattia e ciò può modificare il suo punto di vista relativamente a decisioni prese nel passato. Si mette alla prova anche l’adattamento della persona a nuove condizioni di vita.
- Tiene conto di teorici significativi avanzamenti della medicina.
- *«In ogni caso i medici sono coloro che assicurano che il processo decisionale sia propriamente condotto e, in particolare, che ogni desiderio precedentemente espresso dal paziente sia tenuto in considerazione e che ogni trattamento inutile o sproporzionato sia evitato»* (Consiglio d’Europa 2014)

“Sono differenti persone?”

Eugenio nel 2013
neodiagnosticato

Eugenio nel 2023
In fase di demenza

progressione della demenza

Parfit D. Reasons and persons. Oxford University Press 1984.
Dworkin R. Life’s dominion. An argument about abortion and euthanasia London:Harper Collins Publishers, 1993.

Demenza e identità personale

Rebecca Dresser / Derek Parfit:

- “Ci sono differenti persone”: il malato può diventare una persona diversa da quella che era prima che si manifestasse la demenza.
- Se l’identità personale è condizione necessaria per la responsibilità morale, allora la persona precedente non ha più nessun obbligo morale sulla persona dopo, che può avere volontà diverse da quelle precedentemente affermate.
- Questa visione rende praticamente le direttive anticipate prive di autorità morale.

Ronald Dworkin:

- Esiste una vita psicologica che viene progressivamente spenta dalla malattia: il passaggio graduale dalla competenza alla sua completa perdita si inscrive in una singola esperienza di vita.
- La persona con demenza è la stessa persona alla quale però la malattia ha modificato o annullato l’identità personale: non c’è una persona “demente”, c’è una persona che “è diventata demente”.
- Tale richiamo all’identità pre-morbosa rende valida qualsiasi decisione presa prima della malattia.

Dresser, R. Dworkin on dementia. Hastings Center Report 25:32–8, 1995.
Parfit D. Reasons and persons. Oxford University Press 1984.
Dworkin R. Life’s dominion. An argument about abortion and euthanasia London:Harper Collins Publishers, 1993.

Ruolo del fiduciario

- Per chi sta affrontando un declino cognitivo, la disponibilità di un fiduciario che ha condiviso, almeno in parte, la sua vita e possa rappresentare la sua biografia, può costituire ancora di più una garanzia della propria identità di fronte alle scelte da compiere oggi per il futuro.

Una moglie che parla del proprio marito affetto da demenza:

- …se mi avessero detto 10 ann ifa, quando la malattia è iniziata, che in 10 anni mio marito sarebbe diventato immobile, senza possibilità di parlare, incontinente e completamente dipendente... E qualcuno mi avesse chiesto “C’è qualità di vita in un tale stadio di malattia?’ Penso che 10 anni fa avrei detto, ‘No’.
- Ma ora … gli piace il calore del sole sulle mani, ... gli piace la sua musica ... ama il contatto umano... E sì, c’è ancora qualità di vita...

Hughes, J.C. and Baldwin, C. (2006) *Ethical Issues in Dementia Care: Making Difficult Decisions. London: Jessica Kingsley.*

Alcune barriere che considero più specifiche in presenza di disturbi cognitivi

- Maggiore difficoltà ad obiettivare iniziali disturbi cognitivi edincertezza prognostica relativa alla loro evoluzione

- Ne deriva difficoltà a stabilire il *timing* giusto per proporre una PCC relativamente al «*trigger*» cognitivo

- *"…physicians observe that patients with dementia themselves often prefer not to initiate such discussions about future care, because they are unaware of the diagnosis and prognosis or because of diminished decision-making capacity"*

Keijzer-van Laarhoven AJJM, et al. BMJ Open 2020;10:e038528. doi:10.1136/bmjopen-2020-038528

Alcune barriere che considero più specifiche in presenza di disturbi cognitivi

Katsari, M., Kasselimis, D. S., Giogkaraki, E., Breza, M., Evangelopoulos, M. E., Anagnostouli, M., Andreadou, E., Kilidireas, C., Hotary, A., Zalonis, I., Koutsis, G., & Potagas, C. (2020). A longitudinal study of cognitive function in multiple sclerosis: is decline inevitable?. Journal of neurology, 267(5), 1464–1475. https://doi.org/10.1007/s00415-020-09720-8

Eijlers, A. J. C., van Geest, Q., Dekker, I., Steenwijk, M. D., Meijer, K. A., Hulst, H. E., Barkhof, F., Uitdehaag, B. M. J., Schoonheim, M. M., & Geurts, J. J. G. (2018). Predicting cognitive decline in multiple sclerosis: a 5-year follow-up study. Brain : a journal of neurology, 141(9), 2605–2618. https://doi.org/10.1093/brain/awy202

Alcune barriere che considero più specifiche in presenza di disturbi cognitivi

- Maggiore difficoltà ad obiettivare iniziali disturbi cognitiviedincertezza prognostica relativa alla loro evoluzione
- Ne deriva difficoltà a stabilire il *timing* giusto per proporre una PCC relativamente al solo «*trigger*» cognitivo (problema poco frequente)
- *"…physicians observe that patients with dementia themselves often prefer not to initiate such discussions about future care, because they are unaware of the diagnosis and prognosis or because of diminished decision-making capacity"*
- *"Especially when patients do not initiate the conversation themselves, physicians may regard taking the initiative as inappropriate. This uncertainty about who should start the discussion can make physicians reluctant to assume responsibility"*

Sellars et al. Perspectives of people with dementia and carers on advance care planning and end-of-life care: A systematic review and thematic synthesis of qualitative studies. Palliat Med. 2019 Mar;33(3):274-290.

Keijzer-vanLaarhovenAJJM,etal.BMJOpen2020;10:e038528. doi:10.1136/bmjopen-2020-038528

Dimensione collegiale?

chrome-extension://efaidnbmnnnibpcajpcglclefindmkaj/https://rm.coe.int/168039e8c2

- Il processo di decisione assume una dimensione collegiale quando il paziente non vuole o non è in grado di parteciparvi direttamente. Quando il paziente non desidera, non può o non è più in grado di partecipare alla decisione o esprime il bisogno di essere sostenuto nel processo, la deliberazione collegiale:
  - dovrebbe fornire delle garanzie se le decisioni sono prese da terzi;
  - dovrebbe adeguarsi alle situazioni e alle scelte complesse emergenti da una situazione di fine vita.
- Il processo di deliberazione collegiale nelle situazioni di fine vita è costituito, di principio, da tre fasi principali:
  - una fase individuale: ogni soggetto nel processo decisionale modula i suoi argomenti sulla base delle informazioni raccolte sul paziente e sulla sua malattia;
  - una fase collegiale: i diversi attori - famiglia, amici prossimi e professionisti sanitari – si consultano e discutono tra loro, fornendo prospettive diverse e visioni complementari (Familiari, amici e figure di supporto sono consultate, a condizione che non vi sia un'obiezione precedente espressa dal paziente);
  - una fase conclusiva: si prende la decisione effettiva.

Comitato di Bioetica del Consiglio d'Europa (DH-BIO) - 2014

"In essenza la medicina è una attività a valenza morale fondata su un patto fiduciario"

Crawshaw et al. Patient-physician covenant, JAMA, 1995, 273:155

Proprio la posizione del paziente incapace, che non abbia espresso disposizioni anticipate di trattamento o non abbia pianificato in modo condiviso le cure, pone infatti i problemi più delicati in ordine, da un lato, al rispetto della sua capacità di autodeterminazione e, dall'altro, all'erogazione delle cure più appropriate e ragionevoli … (Noccelli, 2018)

ConCure-SM

Training dei Professionisti Sanitari

AmadeoLab, 15-16 ottobre 2021

PIANIFICAZIONE CONDIVISA DELLE CURE

Marta Cascioli

Usl Umbria 2 Hospice La Torre sul Colle Spoleto

LA MIA PIANIFICAZIONE CONDIVISA DELLE CURE

Lemiesceltedicura rispettoallamiasaluteealfinevita

-Nel 2050 più di un terzo della popolazione dell'UE avrà più di 60 anni;
13 milioni di multicronici 2024, cioè oltre il 20% della popolazione.

Walter Ricciardi, il Servizio Nazionale rischia il collasso? L'arco di Giano N 89/2016

MUTAZIONE EPIDEMIOLOGICA E DEMOGRAFICA

Christensen, K., Doblhammer, G., Rau, R., & Vaupel, J. W. (2009). Ageing populations: the challenges ahead. Lancet (London, England), 374(9696), 1196–1208. https://doi.org/10.1016/S0140-6736(09)61460-4

Tra le patologie cronico-degenerative c'è una prevalenza di quelle neurologiche, con persone con intensi bisogni di cura.

JAgingSocPolicy.2016Jul-Sep;28(3):218-31.doi: 10.1080/08959420.2016.1181972.
Meeting the Needs of theGrowing VeryOld Population: Policy Implications for a Global Challenge.
Pin S1,Spini D1.

LE CURE PALLIATIVE NEL MALATO NEUROLOGICO

Magnani, C., Peruselli, C., Tanzi, S., Bastianello, S., Bonesi, M. G., Moroni, L., & Orsi, L. (2019). Complessità e cure palliative. Rivista Italiana di Cure Palliative, 21(3), 196-203. https://doi.org/10.1726/3225.32031

Grant, M., de Graaf, E., & Teunissen, S. (2021). A systematic review of classifications systems to determine complexity of patient care needs in palliative care. Palliative medicine, 35(4), 636–650. https://doi.org/10.1177/0269216321996983

"**SURPRISE QUESTION**":

"quando visiti un malato, saresti sorpreso se morisse nei prossimi 12 mesi a causa della malattia neurologica o delle sue complicanze? .

Se larisposta è 'no ' dai la precedenza alle sue preoccupazioni, al controllo dei sintomi, all'aiuto alla famiglia, alla continuità delle cure, al supporto spirituale. Per eliminare paure e ansie, i malati cronici devono capire cosa sta accadendo.

Downar, J., Goldman, R., Pinto, R., Englesakis, M., & Adhikari, N. K. (2017). The "surprise question" for predicting death in seriously ill patients: a systematic review and meta-analysis. CMAJ : Canadian Medical Association journal = journal de l'Association medicale canadienne, 189(13), E484–E493. https://doi.org/10.1503/cmaj.160775

LE CURE PALLIATIVE NEL MALATO NEUROLOGICO

BIOTESTAMENTO, LA LEGGE È IN GAZZETTA

ART.1 CONSENSO INFORMATO

COMMA 1

RELAZIONE DI CURA E DI FIDUCIA

ART. 1 COMMA 2

DIRITTO DI INFORMAZIONE
DIRITTO DI NON SAPERE

ART 1 COMMA 3

DIRITTO DI RIFIUTARE DIRITTO DI REVOCA

ART 1 COMMA 5

RISPETTO VOLONTA'

ART. 1 COMMA 6

TEMPO

ART. 1 COMMA 8

ART 1 COMMA 9-10

DISPOSIZIONI ANTICIPATE DI TRATTAMENTO

DISPOSIZIONI ANTICIPATE DI TRATTAMENTO

ART. 5 PIANIFICAZIONE CONDIVISA DELLE CURE

PIANIFICAZIONE CONDIVISA DELLE CURE

ART 5 COMMA 2

PIANIFICAZIONE CONDIVISA DELLE CURE

ART 5 COMMA 3-4

VANTAGGI PCC

Riduzione **stress**operatori sanitari
Diminuzione**contenzioso**legato alla responsabilità medica
Riduzione tassi **ospedalizzazione impropri**
Appropriatezza e diminuzione **spesa**sanitaria
Miglioramento **qualità**dell’assistenza
Aumento **qualità**vita residua

Johnson, S., Butow, P., Kerridge, I., & Tattersall, M. (2016). Advance care planning for cancer patients: a systematic review of perceptions and experiences of patients, families, and healthcare providers. Psycho-oncology, 25(4), 362–386. https://doi.org/10.1002/pon.3926

Tolle, S. W., Back, A. L., & Meier, D. E. (2015). Clinical decisions. End-of-life advance directive. The New England journal of medicine, 372(7), 667–670. https://doi.org/10.1056/NEJMclde1411152

Dube, M., McCarron, A., & Nannini, A. (2015). Advance care planning complexities for nurse practitioners. Journal for Nurse Practitioners, 11(8), 766–773. https://doi.org/10.1016/j.nurpra.2015.05.011

Brinkman-Stoppelenburg, A., Rietjens, J. A., & van der Heide, A. (2014). The effects of advance care planning on end-of-life care: a systematic review. Palliative medicine, 28(8), 1000–1025. https://doi.org/10.1177/0269216314526272

Detering, K. M., Hancock, A. D., Reade, M. C., & Silvester, W. (2010). The impact of advance care planning on end of life care in elderly patients: randomised controlled trial. BMJ (Clinical research ed.), 340, c1345. https://doi.org/10.1136/bmj.c1345

Gilissen, J., Pivodic, L., Wendrich-van Dael, A., Gastmans, C., Vander Stichele, R., Van Humbeeck, L., Deliens, L., & Van den Block, L. (2019). Implementing advance care planning in routine nursing home care: The development of the theory-based ACP+ program. PloS one, 14(10), e0223586. https://doi.org/10.1371/journal.pone.0223586

chrome-extension://efaidnbmnnnibpcajpcglclefindmkaj/https://www.atlanticare.org/sites/default/files/pdf/advance-care-planning-conversation-starter.pdf

PROGRAMMI E PROGETTI

The Conversation Project. (2016). Our purpose. Retrieved from http://theconversationproject.org/about/

Prandi, C. (2019). Gli infermieri si posizionano rispetto alla Legge 219/2017. Rivista Italiana di Cure Palliative, 21, 231–235. https://doi.org/10.1726/3225.32031

chrome-extension://efaidnbmnnnibpcajpcglclefindmkaj/https://www.fnopi.it/wp-content/uploads/2019/11/Linee-Guida-Fine-Vita.pdf

GLI INFERMIERI E LA LEGGE 219/2017

ART 24 CURA NEL FINE VITA

Art 4 RELAZIONE DI CURA

Nell'agireprofessionalel'infermierestabilisceuna relazionedi cura, utilizzandoanchel'ascoltoe il dialogo.

Si fa garantechela persona assistitanon siamai lasciatain abbandonocoinvolgendo, con il consenso dell'interessato, le sue figure di riferimento, nonchéle altrefigure professionali e istituzionali.

Il tempo di relazioneè tempo di cura.

chrome-extension://efaidnbmnnnibpcajpcglclefindmkaj/https://www.fnopi.it/wp-content/uploads/2025/03/FNOPI_CodiceDeontol2025_web.pdf

In casa avevo tre sedie, una per la solitudine, due per l'amicizia, tre per la compagnia.

Henry David Thoreau

Walden. Vita nei boschi

GRAZIE

ConCure-SM

Training dei Professionisti Sanitari
AmadeoLab, 15-16 ottobre 2021

La pianificazione condivisa delle cure

Ludovica De Panfilis
Unità di Bioetica
Azienda USL-IRCCS di Reggio Emilia

LA MIA PIANIFICAZIONE CONDIVISA DELLE CURE
Lemiesceltedicura rispettoallamiasaluteealfinevita

https://www.la7.it/in-onda/video/afghanistan-il-racconto-di-francesca-mannocchi-il-paese-si-sente-tradito-16-08-2021-393530

«Ho una malattia neurologica cronica. Sono una donna di 39 anni malata di sclerosi multipla. Nel mio caso la malattia è recidivante remittente. Significa che i sintomi a volte peggiorano (le fasi recidive) a volte danno tregua (le fasi remittenti). Perciò, nell’espressione «malattia potenzialmente debilitante del cervello e del midollo spinale» la parola chiave è potenzialmente. Il peggioramento è potenziale, la stabilizzazione è potenziale. L’immobilità è potenziale, la cecità lo è. Dunque, dal primo giorno, ho stabilito che anche la paura sarebbe stata potenziale»

Mannocchi, F. (2021). Bianco è il colore del danno. Einaudi.

Le parole chiave della legge n.219/2017

- Dignità
- Disporre
- Pianificare
- Scelta
- Consenso
- Ostinazione irragionevole
- Rispetto
- Diritto
- Valore
- Testimoniare!

→ Qualità della vita e qualità della morte

Per quali diritti?

1. Diritto alla vita
2. Diritto alla salute
3. Diritto alla dignità
4. Diritto all'autodeterminazione

"Riconoscere il valore in sé di ogni essere umano, e quindi la sua intrinseca dignità, significa rispettarne le scelte, i desiderie i valori"

Articolo 2: Terapia del dolore, divieto di ostinazione irragionevole nelle cure e dignità nella fase finale della vita

Nei casi di paziente con prognosi infausta a breve termine o di imminenza di morte, il medico deve astenersi da ogni ostinazione irragionevole nella somministrazione delle cure e dal ricorso a trattamenti inutili o sproporzionati.In presenza di sofferenze refrattarie ai trattamenti sanitari,il medico può ricorrere alla sedazione palliativa profonda continua in associazione con la terapia del dolore, con il consenso del paziente

«L'inviolabile dignità della persona presidia la sua qualità di vita»

Stefano Rodotà

Ma le persone non vogliono parlarne...(?)

De Panfilis, L., Rossi, P. G., Mazzini, E., Pistolesi, L., Ghirotto, L., Noto, A., Cuocolo, S., & Costantini, M. (2020). Knowledge, Opinion, and Attitude About the Italian Law on Advance Directives: A Population-Based Survey. Journal of pain and symptom management, 60(5), 906–914.e4. https://doi.org/10.1016/j.jpainsymman.2020.06.020

Ma le persone non vogliono parlarne...(?)

«Ioso che le mie fratture, le mie placche attive, la diminuzione della velocità di conduzione dei miei segnali elettrici neuronali corrispondono alla possibilità che io possa svegliarmi e non vederci da un occhio, non riuscire a deglutire, non muovere una gamba. Nessuno ti avverte, con queste parole, che possa accadere. Nessuno ti allarma, è vero. Ma nessuno, nemmeno, ti prepara. Nell’ultimo quarto di secolo la relazione verbale con il paziente è diventata più semplice, ci sono meno probabilità di diventare disabile. Va bene, dico. Ma come si comunica ad un paziente che può finire domani, imprevedibilmente, sulla sedia a rotelle?Non si comunica. Eppure, alla domanda: può accadere, giusto? La risposta continua ad essere: in linea di principio, si».

La PCC: parliamo di valori!

“Healthcare professionals and patients often present and discuss treatment options thoroughly. Explicitly or

implicitly, this inherently involves a discussion of values, norms and virtues in order to make good choices”

WiddershovenG, AbmaT, MolewijkB. Empirical ethics as dialogical practice. Bioethics. 2009 May;23(4):236-48. doi: 10.1111/j.1467-8519.2009.01712.x. PMID: 19338524.

La PCC: parliamo di valori!

Il booklet

«Diario, Ottobre 2017: Cose che temo di non poter fare più: Nuotare con mio figlio, fare l'amore, sentire una carezza, dare una carezza, fare la valigia, sfogliare i libri, toccare la sabbia…»

«La PCC è un Processo strutturato multidisciplinareche pone grande enfasi sulle preferenze e i valori del paziente e della famiglia ed incoraggia le discussionirelative ai possibili scenari con il paziente e, nel caso di minore, con i genitori/rappresentanti legali»

La PCC: parliamo di valori!

Il booklet

«Non ho paura della mia disabilità, ho paura che mio figlio abbia una madre disabile. Non mi spaventa tanto la sedia a rotelle, mi spaventa che mio figlio abbia una madre sulla sedia a rotelle. La malattia di uno diventerebbe la malattia di tutti. La disabilità di uno, la disabilità della famiglia. Che fare? Devo configurarmi uno spazio nuovo. Esercizio: provare ad immaginarsi come una madre compromessa»

La PCC: parliamo di valori!

"L'insieme degli obiettivi, delle aspettative, delle personali predisposizionie credenzeche una personapossiede in merito ad una particolare decisione e alle conseguenze di quella decisione"

Guyatt G. et al., 2015

«L'identità è una faccenda complessa e cercarsi può diventare una condanna a vita»

Forte, D. N., Kawai, F., & Cohen, C. (2018). A bioethical framework to guide the decision-making process in the care of seriously ill patients. BMC Medical Ethics, 19(1), 78.

Le «competenze etiche»: di cosa parliamo?

"The concept of ethical competence can be defined in terms of character strength, ethical awareness, moral judgement skills and willingness to do good.

Virtuous professional, experience of a professional, human communication, ethical knowledge and supporting surroundings in the organizationcan be seen as prerequisites for ethical competence.

Ethical competence results in the best possible solutions for the patient, reduced moral distress at work and development and democratisationof society.

Kulju, K., Stolt, M., Suhonen, R., & Leino-Kilpi, H. (2016). Ethical competence: A concept analysis. Nursing ethics, 23(4), 401–412. https://doi.org/10.1177/0969733014567025

Le «competenze etiche»: di cosa parliamo?

De Panfilis, L., Tanzi, S., Perin, M., Turola, E., & Artioli, G. (2020). "Teach for ethics in palliative care": a mixed-method evaluation of a medical ethics training programme. BMC palliative care, 19(1), 149. https://doi.org/10.1186/s12904-020-00653-7

Levati, G. P. (2005). Psicologia e sviluppo delle risorse umane. Milano: FrancoAngeli.

CONOSCENZE: Le conoscenze comprendono l’aspetto delle conoscenze teoriche, che assicurano la comprensione dellarealtà, e le cosiddette skillsintese come possesso di strumenti, tecniche e metodologie che mi permettono di gestire e trovare soluzioni.

CAPACITA’: dotazione personale che permette di eseguire con successo una determinata prestazione. Questa possibilità di riuscita è a sua volta condizionata dall’attitudine.

ESPERIENZA FINALIZZATA: sperimentazione di particolari attività lavorative o extra lavorative, che hanno consentito di esercitare, provare, esprimere le capacità e le conoscenze possedute dalla persona.

COMPETENZA: caratteristica intrinseca dell’individuo, appartenente alla dimensione psicologica, costituita dall’insieme articolato di capacità, conoscenze, esperienze finalizzate. Si esprime attraverso comportamenti.

COMPORTAMENTO: aspetto visibile e concreto della competenza reso possibile dall’insieme delle capacità. Insieme di azioni osservabili, messe in atto da una persona, sollecitate dallacombinazione di contesto e di caratteristiche proprie della persona.

Le «competenze etiche»: di cosa parliamo?

CONOSCENZE
CONOSCERE E LE PRINCIPALI FONTI ETICHE E GIURIDICHE
Conosce l'evoluzione della filosofia e dei codici di deontologia
Conosce l' analisi e la riflessione degli attuali approcci teorici all'etica medica
Conosce e comprende il livello base dei principi etici di riferimento
Conosce e comprende i concetti di dignità, diritti, sofferenza del malato.
Conosce il dibattito attuale in materia di etica relativa all'ambitospecifico
Conosce la legislazione contemporanee in relazione alle normative legate alle cure di fine vita
Conoscenza le raccomandazioni principali delle società scientifiche nazionali (SICP) e internazionali (EAPC) in cure palliative e neurologia

CAPACITA' DEI DISCENTI
SVILUPPO DEL PENSIERO CRITICO ED ANALISI ETICA
E' in grado di riconoscere problemi etici in relazione ai casi clinici analizzati
E' in grado di prendere decisioni su problematiche etiche e di sostenerle sulla base della normativa e dei codici di deontologia
Sa argomentare, di fronte a casi con problemi etici, a quali principi etici si fa riferimento (dilemma etico)
E' in grado di spiegare e giustificare in simulazione, ai pazienti, famigliari o colleghi la decisione presa rispetto ad un trattamento di finevita
È capace di affrontare conflitti di interesse tra pazienti ed i loro parenti
È in grado di sviluppare un ragionamento etico, anche utilizzando strumenti già presenti in letteratura
È in grado di assumersi le responsabilità delle propriedecisioniinambitodifinevita
È in grado di dare informazioni etiche a pazienti e famigliari in ambito di fine vita.
È in grado di elaborare, in simulazione, una adeguata pianificazione con un valido accordo con paziente e famiglia

Le «competenze etiche»: di cosa parliamo?

COMPETENZE
SVILUPPO DI COMPORTAMENTI ETICI
Riconosce i problemi etici nella pratica clinica quotidiana e nel lavoro di squadra
Sa spiegare e giustificare le decisioni prese e condivise, relative alle problematiche etiche del fine vita, pratica clinica quotidiana
Sa agire nella pratica clinica in conformità con la conoscenza della legislazione contemporanea di linee guida e di principietici
CONDURRE CONSULENZE ETICHE
Sa aiutare un paziente a prendere una decisione in accordo con i suoi valori personali
È esperto nel discutere i problemi del fine vita con i pazienti e le loro famiglie
Rispetta sempre la riservatezza, rispetto alle problematiche dei pazienti
Sa gestire il conflitto etiche con pazienti e famiglia nella pratica clinica quotidiana
Sa ottenere un valido consenso alle decisioni legate al fine vita da parte del paziente e della famiglia

Responsabilità morale condivisa

Responsabilità morale del paziente:

E’ autonomo l’agente che ha desideri e valori e che sa renderli effettivi!

Manon solo…

Tali desideri e valori devono essere autenticamente appartenenti all’individuo

=

autogoverno del sé + capacità di auto-

riflessione + competenza non solo cognitiva, ma anche normativa e morale!

Responsabilità morale degli operatori: «Non

basta tutelare l’autonomia del paziente nei contesti di cura assicurandosi l’assenza di interferenze: gli operatori sanitari dovrebbero anche migliorare le condizioni positive che “generano” un ragionamento e un processo decisionale autonomo»

N. Stoljar, Theories of Autonomy, in R.E.

Ashcroft et al, Principles of Health care Ethics, 2016

«Dove finisca il nostro dolore quando è ingabbiato nelle griglie rigide della lingua medica è l'altra grande domanda che mi accompagna dai tempi della diagnosi. Quella lingua è prossima alla malattia, ma non le è fedele. L'aridità delle parole scientifiche altera il mio male, lo anestetizza»

Thank you!

ConCure-SM

Training dei Professionisti Sanitari

AmadeoLab, 15-16 ottobre 2021

ADVANCE CARE PLANNING

S. Veronese

LA MIA PIANIFICAZIONE CONDIVISA DELLE CURE

Lemiesceltedicura rispettoallamiasaluteealfinevita

SCLEROSI MULTIPLA associazione italiana
un mondo libero dalla SM

Fondazione I.R.C.C.S. Istituto Neurologico Carlo Besta

Sistema Socio Sanitario Regione Lombardia

Manuale di utilizzo

Il booklet è indirizzato alla PcSM e questo può erroneamente portare a pensare che i curanti non ne siano i destinatari. In realtà, la PCC è un processo che avviene esclusivamente nella relazione tra paziente e medico, come descritto al comma 1, Art. 5 della Legge: “[…] può essere realizzata una pianificazione delle cure condivisa tra il paziente e il medico, alla quale il medico e l'equipe sanitaria sono tenuti ad attenersi qualora il paziente venga a trovarsi nella condizione di non poter esprimere il proprio consenso o in una condizione di incapacità.

Il booklet dunque diventa uno strumento di cura, che sarà proposto dal medico di fiducia (il neurologo del centro SM nel caso del progetto ConCure-SM, ma anche il MMG, il medico palliativista, in accordo e con la collaborazione degli altri membri dell’equipe curante possono solitamente proporre questo strumento) alla PcSM in previsione di una possibile perdita di capacità decisionale della stessa.

Contenuti:

1. La mia pianificazione condivisa delle cure
2. Cosa è importante per me
3. Cosa mi preoccupa
4. Perché voglio fare una ‘Pianificazione Condivisa della Cure’
5. Come prendo le decisioni
6. Se non fossi più in grado di decidere: il mio fiduciario
7. Pensando alla fine della mia vita
8. Le mie scelte di cura
9. Firme
10. Abbreviazioni

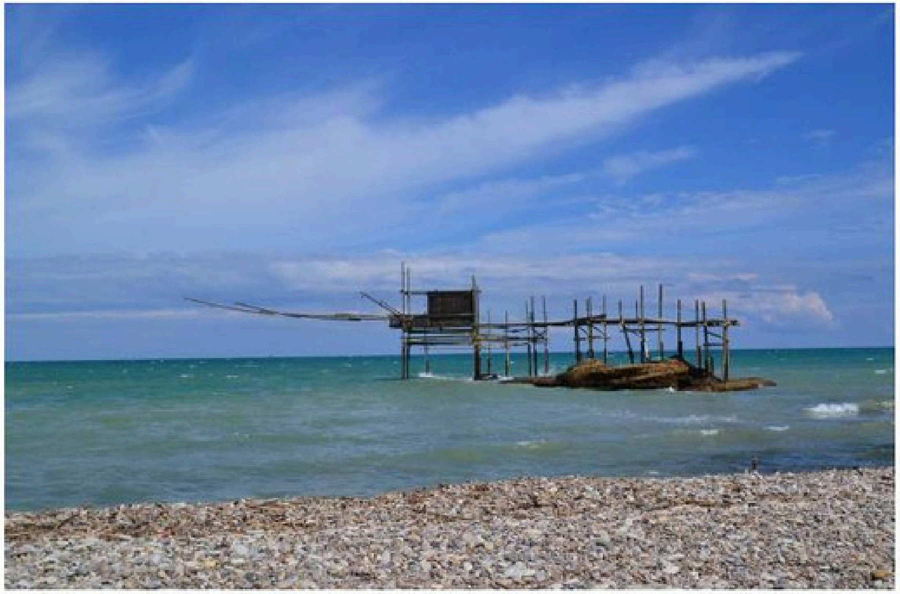

1. La mia pianificazione condivisa delle cure

Questa è la mia Pianificazione Condivisa delle Cure e contiene le mie scelte.

Per favore, seguitela qualora non fossi più in grado di esprimere quello che desidero.

Nome		Cognome	
nato/a il		a	
CF			
Indirizzo			
Telefono			
e-mail			

2. Cosa è importante per me

Alcune domande che possono aiutarti a definire cosa sia importante per te:

- Cosa ti rende felice?
- Cosa ti reca piacere e gioia?
- Che cosa ti piace fare?
- Quali sono i tuoi hobby e i tuoi interessi?
- Ci sono delle abitudini alle quali sei affezionato?
- Che cosa dà senso alla tua giornata?
- Con chi ti piace trascorrere il tempo?
- Hai principi spirituali, religiosi, o riti che sono importanti per la tua vita?

Ecco alcune altre cose che potrebbero essere importanti o significative per te:

- Parlare e stare vicino alle persone
- Renderti conto di chi sei e dove ti trovi
- Sentire l'amore e l'affetto degli altri
- Vivere esperienze significative
- Avere vicino il cane o l'animale di compagnia
- Partecipare al culto della tua religione
- Sentirti attivo culturalmente
- Contribuire al bene della società
- Sentire che qualcuno ti abbraccia e ti tiene per mano
- Mantenere il più possibile l'autonomia
- Avere momenti di intimità o sessualità

Questo è ciò che voglio che i miei curanti e i miei cari sappiano di me e di cosa è importante per me:

Questi sono i valori (ad esempio culturali, spirituali, religiosi) importanti per me:

Per onorare questi valori desidero che i miei curanti e i miei cari:

3. Cosa mi preoccupa

Ci sono cose che ti preoccupano quando pensi al tuo futuro?

Per esempio, ti preoccupi quando pensi:

- Che la tua salute potrà compromettere le tue scelte
- Che la tua salute potrà causare problemi ai tuoi cari
- Dove sarai assistito in futuro
- Di provare dolore o sofferenza
- Di non essere più in grado di comunicare
- Di perdere la capacità di ragionare
- Di essere di peso per gli altri
- Di venire ricoverato in struttura
- Di morire da solo
- Di come le persone che ami possano andare avanti senza di te
- Di rimanere bloccato in un letto
- Che le tue scelte non siano rispettate
- Che i tuoi valori non siano considerati
- Di avere problemi economici

Questo è ciò che voglio che i miei curanti e i miei cari sappiano rispetto a ciò che mi preoccupa:

Segna le caselle corrispondenti

☐ Soffrire
La sofferenza per me significa:

☐ Non poter comunicare
Ad esempio:

☐ Non poter fare cose
Ad esempio:

☐ Mi preoccupo per i miei cari
Ad esempio perché:

☐ Altre cose
Mi preoccupo di:

4. Perché voglio fare una Pianificazione Condivisa delle Cure

Alcune cose a cui pensare:

- Come è stato l'andamento della tua SM e della tua salute in generale nell'ultimo anno?
- Il tuo stato di salute ti limita fortemente in attività che sono importanti per te?
- Sei aiutato e sostenuto da familiari e, più in generale, da persone care?
- Sei di aiuto e sostegno a familiari e persone care?

Per comprendere meglio che impatto potrà avere il tuo stato di salute sul tuo futuro, parlane con i professionisti sanitari che si prendono cura di te.

Per esempio, potresti chiedere loro: se la mia SM dovesse peggiorare...

- Che livello di indipendenza e autonomia potrò avere?
- Cosa è bene/giusto pianificare ora?
- Cosa accadrà al mio corpo e alla mia mente?
- Che impatto potrebbe avere il mio stato di salute sulle persone che si prendono cura di me?

Ecco perché voglio fare una PCC:

Se penso al mio futuro mi viene in mente:

Se penso al mio futuro mi sento:

Se il tempo davanti a me fosse breve allora vorrei:

5. Come prendo le decisioni

Pensa alle decisioni che potresti dover prendere nel corso della malattia.

Pensa a come sei abituato a prendere le decisioni.

Hai bisogno di tempo? Ti piace essere molto informato sulle possibilità di scelta, o preferisci che siano altri a decidere per te?

Hai mai pensato che nella vita possano verificarsi eventi improvvisi, come incidenti o eventi acuti, in cui debbano essere prese rapidamente delle decisioni importanti?

Chi vorresti che parlasse a nome tuo se tu non fossi in grado di farlo?

Ricorda che, qualora non fossi più in grado di esprimerti, altri dovranno decidere per te. Prenditi dunque del tempo per riflettere e per parlare di questo con le persone che ti sono vicine.

Se decidi di nominare una persona come tuo fiduciario, perché pensi che possa rappresentare adeguatamente il tuo punto di vista nelle decisioni che riguardano la tua salute, potrebbe essere il momento giusto per farlo.

Potrai revocare questa scelta in ogni momento.

Il tuo fiduciario parlerà a nome tuo solo nel caso tu non possa esprimere la tua preferenza.

Segna la casella che più corrisponde alla tua preferenza

Voglio avere...

	①	②	③	④	⑤	
Solo le informazioni strettamente necessarie	☐	☐	☐	☐	☐	Tutti i dettagli sulla mia malattia e le terapie

Voglio che i miei curanti...

	①	②	③	④	⑤	
Facciano quello che pensano sia meglio per me	☐	☐	☐	☐	☐	Mi consentano di dire la mia in ogni circostanza

Se la mia SM raggiungesse una fase avanzata vorrei...

	①	②	③	④	⑤	
Sapere quanto mi resta da vivere	☐	☐	☐	☐	☐	Non sapere quanto mi resta da vivere

Voglio che i miei cari...

	①	②	③	④	⑤	
Decidano rispettando esattamente la mia volontà, anche se questo li facesse stare male	☐	☐	☐	☐	☐	Prendano la decisione che li faccia sentire in pace, anche se dovesse essere contraria alla mia volontà

Voglio che i miei cari...

	①	②	③	④	⑤	
Non sappiano nulla sul mio stato di salute	☐	☐	☐	☐	☐	Ricevano ogni informazione sul mio stato di salute

6. Se non fossi più in grado di decidere: il mio fiduciario

Se hai deciso di nominare un fiduciario, devi coinvolgerlo nelle tue scelte future.
Parla con il fiduciario del tuo piano di cure e consegnagli, quando lo avrai compilato, una copia del documento di PCC.

Se non hai ancora pensato a un fiduciario, prova a valutare se non sia il caso di farlo ora.
Per scegliere la persona, o le persone, che dovranno decidere per la tua salute nel momento in cui tu non fossi più in grado di farlo, pensa a qualcuno che:

- Ti conosca bene
- Si preoccupi di cosa è importante per te
- Sia disponibile a parlare di questi aspetti con te
- Ti ascolti e sia rispettoso delle tue scelte
- Sia disposto a difendere le tue volontà affinché vengano esaudite

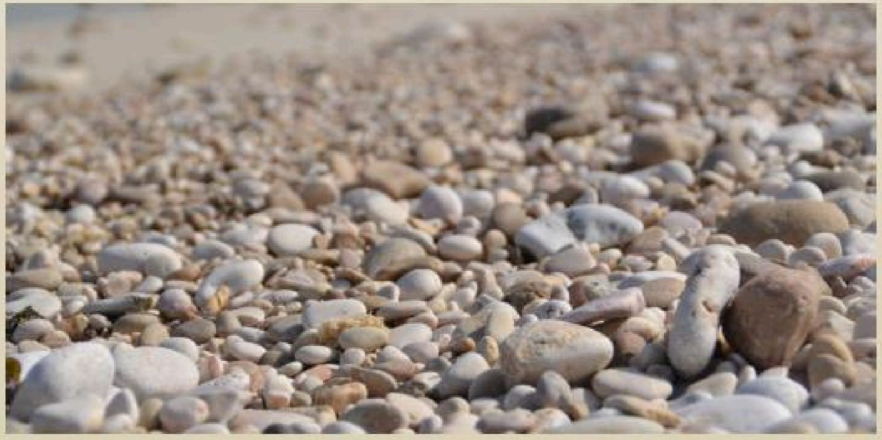

Se perdessi la capacità di decidere:

Segna la casella che corrisponde alla tua preferenza

☐ Ho scelto il fiduciario di seguito indicato

Nome e cognome ____________________

Indirizzo ____________________

Telefono ____________ e-mail ____________

• *Se il mio fiduciario fosse impossibilitato a svolgere il suo ruolo, indico come seconda persona di fiducia*

Nome e cognome ____________________

Telefono ____________ e-mail ____________

• *Come terza persona di fiducia*

Nome e cognome ____________________

Telefono ____________ e-mail ____________

☐ Non ho scelto un fiduciario

Vorrei comunque che la persona di seguito indicata sia informata dai sanitari che prenderanno decisioni sulle mie cure future in base alle indicazioni contenute in questo documento ed in funzione del mio migliore interesse.

Nome e cognome ____________________

Indirizzo ____________________

Telefono ____________ e-mail ____________

7. Pensando alla fine della mia vita

Morire è parte del vivere, ma ci preoccupa e spaventa.
È desiderabile che la fine della vita avvenga nel rispetto della propria dignità e autonomia, in un luogo adeguato e possibilmente di nostra scelta, in presenza delle persone a noi care, se lo vogliamo, e limitando ogni tipo di sofferenza. Non esiste un percorso uguale per tutti alla fine della vita, esso infatti può essere influenzato dall'età, dalle malattie di cui soffriamo e da altre circostanze. In questa fase, potrebbe essere necessario ricevere farmaci e trattamenti con l'obiettivo di controllare sintomi che possono presentarsi quali dolore, mancanza di fiato, nausea, ansia, agitazione. Nei rari casi nei quali la sofferenza non fosse gestibile con terapie ordinarie potrebbe essere indicata una sedazione palliativa profonda, ovvero un trattamento che annulla gradualmente la coscienza, con lo scopo di ridurre la sofferenza sino al sopraggiungere della morte (la sedazione palliativa profonda, infatti, non anticipa né procrastina il momento della morte).

Pensando a cosa significhi per te mantenere una buona qualità della vita, in questa fase cosa credi che sarebbe importante?

- Restare vigile e mantenere il controllo il più a lungo possibile
- Non sentire alcuna sofferenza anche a costo di essere sonnolento o addormentato
- Avere accanto chi amo
- Stare da solo

Dovendo pensare alla fine della tua vita:
- Quale sarebbe la tua morte ideale?
- Pensando alla morte ed al morire, cosa ti preoccupa di più?
- Chi vorresti avere accanto?
- Che tipo di assistenza spirituale o religiosa vorresti?
- In prossimità della morte, cosa vorresti e cosa non vorresti?

Per me una buona qualità della vita in prossimità della morte significa:

Vorrei anche aggiungere:

Quando starò morendo desidero essere curato e accudito nel rispetto della mia persona e della mia dignità. Inoltre desidero:

Segna la casella/le caselle che corrispondono a ciò che desideri

☐ Che vengano interrotti trattamenti non più utili

☐ Mantenere il contatto con le persone che mi sono care

☐ Avere un sostegno spirituale o religioso

Manuale di utilizzo pag.3

Dove vorresti trascorrere le tue ultime settimane o giorni?
- Cosa ritieni necessario affinché questo possa avvenire?

Chi dovrà essere informato del fatto che stai per morire?
- Dove conservi i contatti (nome, telefono) di queste persone?
- C'è qualcuno che potrà contattarle?

Nel caso non fosse possibile soddisfare la tua scelta sul luogo dove morire, hai altre preferenze da esprimere?

Quali altre cose sarebbero importanti per te? (Per esempio, mantenere la tua privacy, ascoltare una musica particolare, poter vedere alcune persone significative, ecc.)

Per me il luogo dove morire...

☐ **È importante** ☐ **Non è importante**

Quando starò morendo vorrei essere assistito:

- ☐ A casa
- ☐ In ospedale
- ☐ In una struttura (comunità, casa di riposo)
- ☐ In hospice
- ☐ Non è rilevante il luogo dove sarò assistito
- ☐ Altro

Che per me significa:

Altri aspetti che vorrei venissero considerati:

8. Le mie scelte di cura nel fine vita

Questa parte del documento va compilata con l'aiuto del tuo medico di fiducia.

I trattamenti di supporto vitale possono mantenerti in vita nelle stesse condizioni in cui ti trovi ora. Altre volte essi possono consentire condizioni di vita per te inedite e difficili da immaginare, o risultare fastidiosi o dolorosi. Tra questi trattamenti vi sono l'idratazione/nutrizione 'artificiale' (per sondino naso-gastrico, PEG, per via parenterale/endovenosa), la rianimazione cardiopolmonare (RCP), la ventilazione meccanica con o senza tracheostomia, la dialisi. I trattamenti di supporto vitale, in sé, non sono né buoni né cattivi, dipende da come e quando vengono utilizzati. È importante, inoltre, ricordare che questi trattamenti non devono essere considerati irreversibili e che si può tornare indietro anche in queste scelte.

Puoi decidere se ricevere, o meno, questi trattamenti. I tuoi curanti ti proporranno solo trattamenti utili per la tua condizione, come per esempio la RCP, che potrebbe riattivare la funzione del cuore o dei polmoni. In tal caso, sei chiamato a decidere se vuoi che venga fatta o meno.

Pensa a cosa è importante per te. Per esempio, la qualità della tua vita (non soffrire) o la durata della tua vita (poter vivere il più a lungo possibile). La tua PCC serve in particolare nelle condizioni di emergenza ove tu non sia in grado di prendere delle decisioni per facilitare i curanti a mettere in atto, o meno, trattamenti nel tuo miglior interesse. Trattamenti appropriati sul piano strettamente tecnico, potrebbero infatti essere inappropriati alla luce delle tue preferenze.

Ci sono circostanze nelle quali non vorresti essere mantenuto in vita e preferiresti non iniziare o sospendere terapie di supporto?

Se mi trovassi in condizioni di estrema gravità, in pericolo di vita ed incapace di decidere per me, ciò che segue descrive al meglio le mie preferenze di cura. Sono consapevole di non poter pretendere trattamenti che i medici giudichino inappropriati per le mie condizioni.

Estrema gravità per me significa:

Ho depositato le mie **Disposizioni Anticipate di Trattamento,** presso il Comune di: [ ]

in data: [ ]

e sono reperibili presso il Registro Nazionale DAT.

Questo documento aggiorna le mie DAT

ATTENZIONE! Da compilare solo se hai già redatto le DAT

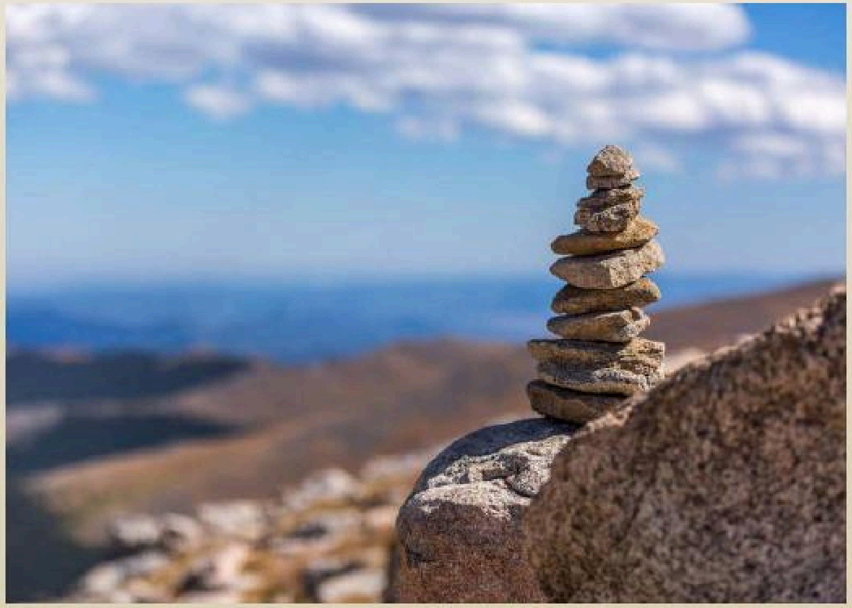

*Scrivi nel riquadro in fondo alla pagina il numero corrispondente alla **tua scelta di cura** e, dove applicabile, indica con un segno i trattamenti specifici (scelta 2).*

1 Vorrei ricevere tutti i trattamenti disponibili ritenuti necessari e appropriati dai medici che mi cureranno, per mantenermi in vita il più a lungo possibile.

2 Vorrei ricevere solo quei trattamenti mirati non solo a prolungare, ma anche a preservare una qualità di vita ancora accettabile per me. Nello specifico, accetto di ricevere i seguenti trattamenti:

- ☐ idratazione/nutrizione per sondino naso-gastrico
- ☐ PEG
- ☐ idratazione/nutrizione parenterale/endovenosa
- ☐ rianimazione cardiopolmonare
- ☐ ventilazione meccanica senza tracheostomia
- ☐ ventilazione meccanica con tracheostomia
- ☐ dialisi

3 Vorrei ricevere solo le cure mirate al controllo dei sintomi e al mio comfort, nel rispetto della mia dignità. Non voglio alcun trattamento finalizzato solo a prolungare la mia vita.

4 Non sono in grado di decidere adesso. Delego i medici che mi cureranno a prendere le decisioni migliori per me, tenendo in considerazione il parere delle persone che ho indicato nella sezione 6.

Ho scelto l'opzione numero: [ ]

9. Firme

Questa sezione va compilata in ogni sua parte e la firma di questo documento è necessaria affinché esso sia ritenuto valido e sia applicato. Se non puoi firmare, è sufficiente una videoregistrazione in cui i sanitari leggeranno le sezioni 6, 7 e 8 del documento e registreranno le tue scelte.

Se hai nominato un fiduciario, è necessaria anche la sua firma.

Anche il tuo medico di fiducia ed eventuali altri professionisti sanitari che ti hanno in cura devono firmare. Questo per garantire che la PCC sia avvenuta in modo informato e condiviso.

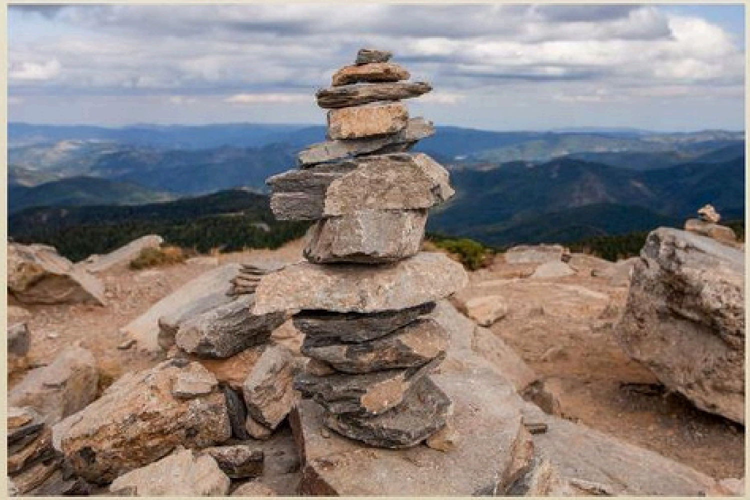

Firmando questo documento io confermo:

1 Di avere compreso la finalità dello stesso e che esso rispecchia le mie volontà

2 Di averlo compilato in piena libertà e dopo essere stato adeguatamente informato

3 Di acconsentire alla custodia delle informazioni di questo documento nei registri, nelle cartelle cliniche e nei fascicoli elettronici previsti, secondo la normativa sulla privacy (Regolamento UE 2016/679) e relativa normativa italiana di adeguamento (D.Lgs. n. 196 del 30 Giugno 2003, così come modificato dal D.Lgs. n. 101 del 10 Agosto 2018).

Nome e cognome ______

Data ______ Firma ______

☐ Non ho scelto un fiduciario

☐ Ho scelto un fiduciario:

Nome e cognome ______

Indirizzo ______

Telefono ______ e-mail ______

Data ______ Firma ______

Manuale di utilizzo pag.3

Ho condiviso con il mio medico di fiducia questo documento:

Dr			
Telefono		e-mail	
Data		Firma	

E, dove applicabile, con il professionista sanitario:

Dr			
Telefono		e-mail	
Data		Firma	

Annotazioni:

10. Abbreviazioni

DAT: Disposizioni anticipate di trattamento
PCC: Pianificazione condivisa delle cure
PEG: Gastrostomia percutanea endoscopica
RCP: Rianimazione cardiopolmonare
SM: Sclerosi multipla

Autori: Michela Bruzzone[1], Marta Cascioli[2], Ludovica De Panfilis[3], Andrea Giordano[4], Maria Grazia Grasso[5], Alessandra Lugaresi[6], Luisa Motti[7], Emanuela Pelle[8], Eugenio Pucci[9], Alessandra Solari[4], Claudio Solaro[10], Simone Veronese[8]

1. Associazione Italiana Sclerosi Multipla, Genova
2. Hospice 'La Torre sul Colle', Spoleto (PG), Azienda USL Umbria 2
3. Azienda USL-IRCCS di Reggio Emilia, Reggio Emilia
4. Fondazione IRCCS Istituto Neurologico Carlo Besta, Milano
5. Fondazione Santa Lucia IRCCS, Roma
6. Dipartimeno di Scienze Biomediche e Neuromotorie, Università di Bologna
7. Hospice 'Casa Madonna dell'Uliveto', Albinea (RE)
8. Fondazione F.A.R.O. Onlus, Torino
9. UOC Neurologia, ASUR Marche, AV4, Fermo
10. CRRF M. L. Novarese, Moncrivello (VC)

Questo opuscolo fa parte del Progetto ConCure-SM; è la traduzione e adattamento di uno strumento di PCC prodotto dalla National ACP programme for New Zealand, 021 928581 Health Quality & Safety Commission.

Realizzazione grafica a cura di Andrea F. Vitali-Studio Manzoni
con il contributo dell'Associazione Marchigiana Sclerosi Multipla e altre Malattie Neurologiche e della Fondazione Italiana Sclerosi Multipla

Foto a cura di: Chiara Uncini. Le immagini di pagina 26, 28 sono tratte da internet. Se il loro utilizzo violasse eventuali diritti d'autore si prega di comunicarlo a: uo-epidemiologia@istituto-besta.it

ConCure-SM

Training dei Professionisti Sanitari
AmadeoLab, 15-16 ottobre 2021

GO WISH

LudovicaDe Panfilis
AziendaUSL-IRCCS diReggio Emilia

LA MIA PIANIFICAZIONE CONDIVISA DELLE CURE

Lemiesceltedicura rispettoallamiasaluteealfinevita

SCLEROSI MULTIPLA associazione italiana
un mondo libero dalla SM
Sistema Socio Sanitario
Fondazione I.R.C.C.S. Istituto Neurologico Carlo Besta
Regione Lombardia

Il gioco di carte Go Wish sviluppato dall'associazione californiana ***Coda Alliance*** è uno strumento finalizzato a stimolare la riflessione e la comunicazione tra

paziente, curantee/o familiare, sullescelterelative al

propriopercorsodi cura.

ABBIAMO VALIDATO E ADATTATO culturalmente il ***Go Wish*** al contesto italiano

(***Protocol number***: 2020/0109816 del 24.09.2020)

Il processo

•Traduzione dall'inglese all'italiano,ad opera di due componenti del gruppo di ricerca.
•Confrontotra le traduzioni e produzione della prima versione italiana (T1) •Traduzione del T1 in inglese ad opera di una traduttrice madrelingua inglese (T2).
Confronto delle traduzioni da parte di un gruppo di esperti, ta cui l'autrice dello strumento.
•Modifica della prima versione italiana sulla base dei commenti raccolti(T3)
•Valutazione qualitativadel T3 tramite2 Focus Group

Risultati:

L'iniziale traduzione italiana è stata modicata con:

- frasi meno esplicite sul fine vita; maggior
- chiarezza sull'utilizzo della carta jolly
- modifica di 3 carte nella versione 'positiva' ('essere senza dolore' al posto di 'non avere dolore')

Risultati step V

Sono stati condotti 2 Focus Group (FG) I FG: sono stati arruolati 8 professionisti (6 medici, 1 psicologa e 1 infermiera); II FG: sono stati arruolati 5 rappresentanti delle associazioni dei pazienti attive sul territorio

L'analisi tematica condotta ulle trascrizioni dei FG ha identificato i seguenti temi:

- Chiarire aspetti linguistici e culturali per usare meglio lo strumento
- Bisogni e suggerimenti per usare il Go Wish nella pratica quotidiana
- Molto più di un gioco'

"Come strumento mi sembra molto interessante e con un'interessante applicazione per il paziente. (...) è un modo per mettersi in gioco, perché è una cosa che normalmente non si fa”

La versionefinale italianadel Go Wish prodotta in questo studio è linguisticamentee culturalmente appropriata al contesto italiano e, rispetto all'originale, contiene3 nuovecarte:

37. Saperecome evolveràla miamalattia;
38. Esserein contattocon le cosebelle dellavita;
39. Sceglierechi mi piacerebbeavere vicino

"Per certi pazienti secondo me può essere utileproprio perchévengono esposti dei pensieri che magari in una semplice comunicazione non salterebbero fuori oppure sarebbe difficile farli emergere in una comunicazione normale, in ambulatorio"

Questa versione potrà facilitare l’implementazione di percorsi di PCC, come previsto dalla L.219/2017.

Accordingto the literature…

Delgado-Guay, M. O., Rodriguez-Nunez, A., De la Cruz, V., Frisbee-Hume, S., Williams, J., Wu, J., Liu, D., Fisch, M. J., & Bruera, E. (2016). Advanced cancer patients' reported wishes at the end of life: a randomized controlled trial. Supportive care in cancer : official journal of the Multinational Association of Supportive Care in Cancer, 24(10), 4273–4281. https://doi.org/10.1007/s00520-016-3260-9

Theten most common very important wishes identified by patients during the first and second tests were:

to be at peace with God

To pray

To have my family with me

To be free from pain

Not being a burden to my family

To trust my doctor

To keep my sense of humor

To say goodbye to important people in my life

To have my family prepared for my death

To be able to help others

Istruzioni

Solitario

1. Legga tutte e 36 le carte e le divida in 3 mazzi:molto importante, abbastanza importante, e non importante per me.

2. Può usare la carta jolly, una o più volte, per affermare qualcosa che desidera e che non è indicata su nessuna delle altre carte. Per esempio: “Essere curato a casa” oppure “Essere in grado di riconoscere la mia famiglia e i miei amici”. Anche questa carta va inserita in uno dei tre mazzi.

3. Quando avrà suddiviso le carte nei tre mazzi, cerchi in quello “molto importante” e ne scelga 10 che descrivono i desideri più importanti per lei. Se non ci sono 10 carte in questo mazzo, ne prenda qualcuna dalla pila “abbastanza importante”.

4. Classifichi le 10 carte dalla più importante alla meno importante. Questo elenco rappresenta la sua “top ten”: le 10 cose più importanti per lei.

Si prenda del tempo per pensare a come vorrebbe spiegare alla sua famiglia o ai suoi amici o ai suoi curanti perché questi desideri rappresentano i suoi desideri più importanti. Pensi anche al suo mazzo di cose non importanti e a come spiegherebbe perché queste cose non sono importanti per lei. Dopo, parli con loro delle sue scelte.

A coppie

Questa modalitàdigiocoèutiledafarecon la persona chevorrebbe diventasseilsuo fiduciario, ovvero la persona che potrebbe rappresentarla in caso lei non fosse più in grado di esprimere le sue volontà. È particolarmente importante che questa persona comprenda i suoi desideri. Sarebbe utile giocare con due mazzi di carte, altrimenti bisognerebbe fare a turno con un solo mazzo.

1. Ogni giocatore legge tutte e 36 le carte. Ogni giocatore le divide in 3 mazzi. Il giocatore A divide le carte in 3 pile: molto importante per me, abbastanza importante per me , non importante per me. Il giocatore B divide le carte in 3 pile, secondo ciò che pensa potrebbe essere importante per il giocatore A: molto importante per il giocatore A, abbastanza importante per il giocatore A, non importante per il giocatore A.

2. I giocatori si confrontano sulle carte nelle tre pile e ciascun giocatore parla di come hanno valutato diversamente le priorità . È molto importante che chiarisca bene le sue scelte. È molto importante che il giocatore B capisca le preferenze del giocatore A.

ConCure-SM

Training dei Professionisti Sanitari

AmadeoLab, 15-16 ottobre 2021

Autoconsapevolezza come strumento di efficacia alla base della buona comunicazione nella pianificazione condivisa delle cure

MichelaRimondini
Universitàdi Verona

LA MIA PIANIFICAZIONE CONDIVISA DELLE CURE

Lemiesceltedicura rispettoallamiasaluteealfinevita

SCLE ROSi MULT iPLA associazione italiana
un mondo libero dalla SM

Fondazione I.R.C.C.S. Istituto Neurologico Carlo Besta

Sistema Socio Sanitario Regione Lombardia

- Obiettivi:

  - Illustrareil ruolo dell’autoconsapevolezza nel processo comunicativo-relazionale.

  - Acquisire alcuni strumenti volti ad aumentarela propria autoconsapevolezzain ambito professionale.

L'atto comunicativo si colloca all'interno di un' interazione dinamica, che è influenzata dalle caratteristiche dei soggetti interagenti e dal contesto in cui ha luogo.

La comunicazione è un atto relazionale multifattoriale che richiede flessibilità e capacità di decentramento.

Nel corso di un’interazione, in poco tempo ci si ritrova a sperimentare specifiche emozioni, sviluppare specifici pensieri e organizzare di conseguenza sulla base di questi elementi un piano d’azione che deve però al contempo tenere conto delle emozioni, credenze, bisogni e informazioni in possesso dell’interlocutore.

Attitudine	Competenza	Abilità	Obiettivo
Autoconsapevolezza	Conoscenze	Tecniche comunicative	Benessere del paziente

Saper fare e saper essere

La professionalità dell'operatore sanitario si fonda su conoscenze, tecniche e abilità che per essere appropriatamente poste in atto devono incardinarsi in una corretta attitudine e piena consapevolezza delle implicazioni personali e professionali legate al ruolo che gioca nella vita della persona che state assistendo.

L'autoconsapevolezza in particolare viene intesa come la capacità del clinico di

osservareericonoscereglielementipersonali,individualichepossonointervenirenel

modulare lo scambio comunicativo.

REFLECTIVE PRACTICE

Novack DH, Suchman AL, Clark W, Epstein RM, Najberg E, Kaplan C. Calibrating the physician.JAMA. 1997;278:502-509

Poiché i medici utilizzano sè stessi come strumento di diagnosi e cura, l'autoconsapevolezza li può aiutarea calibraretale strumentoutilizzandoloin modo piùefficace.

Possiamodefinirel'autoconsapevolezzacome

la comprensione di come le proprie emozioni ed esperienze di vitapossano condizionare l'interazione con I pazienti, familiari e I colleghi

Epstein RM. MindfulPractice.
JAMA.1999;282(9):833–839.
doi:10.1001/jama.282.9.833

Physicians' beliefs and attitudes

Core beliefs/personal philosophy
Family of origin influences
Gender issues
Socio-cultural influences

Physicians' feelings and emotional responses

in patient care
Love, caring, attraction, and boundary setting in medical care
Conflict/anger

Challenging clinical situations

"Difficult patients"
Caring for dying patients
Medical mistakes
Physician self-care

Balancing personal and professional lives

Preventing and managing stress/burnout/impairment

Nel nostro intervento ci focalizzeremo su due aree su cui sviluppare l'autoconsapevolezza in quanto possono esercitare un'importante influenza sulla Comunicazione e PCC:

- Emozioni e stile relazionale
- Sistema valoriale

Autoconsapevolezza e

gestione delle emozioni

e reazioni proprie e

altrui

La teoria dell’attaccamento e i sistemi motivazioni

come strumento per riconoscere e gestire le reazioni dell’operatore sanitario e del paziente al fine di migliorare lo scambio comunicativo

Teoria dell'attaccamento

E' una teoriapsicologica, evoluzionisticaed etologica cheriguardala relazionetra esseriumani, spiegandoin particolarecome il rapporto tragenitoree figlio condizioniil successivo svilupporelazionaledel soggettonellavita adulta.

John Bowlby. A Secure Base: Clinical Applications of Attachment Theory. By. London: Routledge. 1988

In questa teorizzazione il comportamento infantile associato all'attaccamento è principalmente la ricerca della vicinanza a una figura di attaccamento in situazioni stressanti. La qualità delle risposte di tale figura (genitore) porta allo sviluppo di modelli di attaccamento; questi, a loro volta, portano a modelli operativi interni che guideranno i sentimenti, i pensieri e le aspettative dell'individuo nelle relazioni successive. Questi modelli possono essere considerati dalle rappresentazioni mentali della propria amabilità e della disponibilità dell'altro verso di noi.

I sistemimotivazionali Interni

«L’elemento della Teoria dell’Attaccamento che maggiormente ha permesso di ampliare gli orizzonti teorici e clinici della successiva psicoterapia, in particolare della psicoterapia cognitiva, è proprio la concettualizzazione di motivazioni innate che spingono alla costruzione di legami interpersonali e che guidano la costruzione dei significati personali allo scopo di adattarsi all’ambiente attraverso relazioni intersoggettive.» (Farina, Liotti, 2011).

I sistemi motivazionali Interni

Gilbert P. (1989). Human nature and suffering. London, Erlbaum. Liotti G. (1994/2005). La Dimensione interpersonale della Coscienza. Roma, Carocci.

I sistemi motivazionali interpersonali sono tendenze universali, biologicamente determinate e selezionate su base evolutiva.

Orientano i comportamenti secondo obiettivi specifici e sono strettamente legati all'esperienza emotiva.

EMOZIONI E SISTEMI MOTIVAZIONALI

Le emozioniaccompagnano l'azionedei sistemimotivazionaliinterpersonali e possono essere considerate indicatori di attività.

Ogni specificaesperienzaemotiva può esseremeglio compresa se messa in relazione con il sistema motivazionale interpersonale all'interno del quale si colloca.

Ricapitoliamo prima di proseguire…

I sistemi motivazionali sono:

- Sono i principi organizzatori delle interazioni sociali, ovvero rappresentano insiemi di regole interne che guidano il comportamento del soggetto

- Sono disposizioni innate universalmente presenti negli esseri umani

- Sono orientati ad una meta

- Sono attivati da specifici segnali

Ricapitoliamo prima di proseguire…

Quando ci si trova ad interagire con una persona, a seconda di ciò che quell'interazione ci trasmette e del modo in cui lo interpretiamo, assumeremo uno specifico assetto interno che porterà alla messa in atto di specifici comportamenti (verbali e non - comunicazione) nei confronti del nostro interlocutore. Trattandosi si una interazione, lo spesso processo avverrà contemporaneamente nel nostro interlocutore.

Entrando più nello specifico, potremmo dire che le emozioni che esperiamo in un'interazione attivano specifici sistemi motivazionali (e quindi regole di interpretazione della realtà- modelli operativi interni) che a quel punto orienteranno le nostre azioni e interpretazioni in funzione dello scopo da conseguire.

Ricapitoliamo prima di proseguire…

Quindi solo se sono consapevole delle mie emozioni potrò

chiedermi se lo scopo verso cui esse mi stanno orientando è appropriato al contesto in cui mi trovo!

IL SISTEMA DELL'ATTACCAMENTO

" sei fragile, cerca protezione, trova qualcuno che si prenda curadite!"

IL SISTEMA DELL'ATTACCAMENTO

META DEL SISTEMA:
conseguimento della vicinanza protettiva di una persona, possibilmente disponibile a fornire conforto e protezione.
VALORE BIOLOGICO:
le popolazioni in cui gli individui rimangono vicini e sanno chiedere aiuto (per la difesa ed il sostentamento) hanno migliori probabilità di sopravvivenza.
È ATTIVATO DA:
1.fatica,dolore fisico e/o emozionale,solitudine;
2. generale percezione di essere vulnerabile a pericoli ambientali, o di non poter soddisfare da soli i bisogni necessari alla sopravvivenza (alimentarsi, proteggersi dal clima sfavorevole, dormire).
È DISATTIVATO DA:
1.conseguimento dellavicinanza protettiva ad una persona
2.protratta impossibilità di conseguire lameta(diventa distacco emozionale,formapatogena).

IL SISTEMA DELL'ACCUDIMENTO

" proteggi, prenditi cura, aiuta la persona che ti appare più fragile e debole di te!"

IL SISTEMA DELL'ACCUDIMENTO

META DEL SISTEMA:
Offrire vicinanza protettiva, tutelare il soggetto più debole
VALORE BIOLOGICO:
Favorire lepossibilità di difesaesostentamentodegli altri individui dellapropria popolazione.
È ATTIVATO DA:

1)segnalidi richiestadi protezioneprovenienti daun conspecifico(attaccamento);
2). Percezionedi difficoltà, fragilitàdi un conspecifico
È DISATTIVATO DA:
1)segnalidi sollievo e sicurezzaprovenienti dal conspecifico

IL SISTEMA ANTAGONISTA

"Difenditi, lotta, aggredisci o fuggi dalla minaccia di pericolo che hai davanti! Proteggi le tue risorse in quanto limitate!"

IL SISTEMA ANTAGONISTA

META DEL SISTEMA:
Definireil rango di dominanza e sottomissione,proteggerele proprierisorse
VALORE BIOLOGICO:
in un gruppo con ranghi sociali definiti e mantenuti nel tempo i litigi e le lotte sono meno frequenti l'efficienza generale del gruppo è superiore e quindi la sua sopravvivenza
È ATTIVATO DA:
1)percezionecheuna risorsaè limitata;
2) segnali mimici di sfida provenienti da un conspecifico (o interpretati come tali)

È DISATTIVATO DA:
1.segnalidi resae sottomissioneda partedell'antagonista;
2.attivazione di altro sistemamotivazionale (ad es.trasformazione dell'agonismo in cooperazioneo accudimento).

IL SISTEMA COOPERATIVO

"unisciti a qualcuno se questo può favorire la possibilità di raggiungere un obiettivo importante o non rischi di perdere le tue risorse primarie!"

IL SISTEMA COOPERATIVO

META DEL SISTEMA:

Raggiungere un obiettivo comune e condiviso

VALORE BIOLOGICO:

Talvolta la collaborazione tra conspecifici permette una efficienza superiore rispetto all'impegno scoordinato dei singoli

È ATTIVATO DA:

1) percezione di un obiettivo comune

2)segnalidi non minacciaagonistica(sorriso)

È DISATTIVATO DA:

1)euforiao rilassamentoper il conseguimento dell'obiettivo

2)attivazione di altri sistemimotivazionali (es.agonistico)

Da quanto esposto ne deriva che:
l'incidente o criticità comunicativo-relazionale sono legati all'attivazione di un sistema motivazionale non funzionale al contesto.

↓

Da cui ne consegue che la soluzione a tale incidente/criticità deriva dalla capacità di ricollocarsi in un sistema motivazionale adeguato.

4PASSAGGI PER GESTIRE

CORRETTAMENTE LA

COMUNICAZIONE CON IL PAZIENTE PRIMA ANCORA DI INIZIARE A PARLARE!

... O SMETTENDO PER UN MOMENTO DI FARLO.

1

RICONOSCERE E NOMIARE LE TUE EMOZIONI

COSA STO PROVANDO?

2

RICONOSCERE QUALE SM E' STATO ATTIVATO E LA SUA APPROPRIATEZZA AL CONTESTO

L'EMOZIONE CHE STO PROVANDO MI AIUTERA' AD ASSISTERE MEGLIO IL PAZIENTE?

QUALE SM E' ATTIVO IN ME? LO SCOPO VERSO CUI MI ORIENTA, E' COERENTE CON IL MIO RUOLO/COMPITO PROFESSIONALE?

3

CAMBIARE

IL SM

DEVO DIRMI QUALCOSA CHE MI RICHIAMI AL MIO VALORE PROFESSIONALE E ATTIVI COSI' UN'ALTRAEMOZIONEEUNALTRO SM!!!

Stimolo di approfondimento

Quali sono le mie affermazioni automotivanti? Ovvero cosa mi dico in genere per ricollocarmi in un sistema motivazionale più funzionale?

4
RICONOSCERE L'EMOZIONE *DEL PAZIENTE*

COSA STA PROVANDO? COME SI SENTE? DI COSA HA BISOGNO?

Solo dopo aver riconosciutoil
proprio vissutoemotivo, siè
valutatala funzionalitàdel
sistemamotivazionalechesiè
attivatoe sisonoindividuati
gli obiettivi appropriati
all’interazione, si potrà
rendererealmenteefficacee
autenticoil proprio
interventocomunicativoed
essere certi che sia centrato
sulbisognodel pazientee
non reattivoal nostro
bisogno inconscio.

Autoconsapevolezza dei propri sistemi valoriali e preferenze nel fine vita

ConCure-SM Fase 2

TrainingResidenzialedegliOperatori
Milano, AmadeoLab

Scenari per la costruzione dei role playing nei laboratori

Introduzione Nel corso del training residenziale mirato alla formazione del personale operante nei centri SM sulla Pianificazione Condivisa delle Cure – PCC- sono previsti cinque laboratori, che saranno svolti attraverso l'utilizzo di role playing di simulazione di situazioni cliniche. L'obiettivo dei laboratori è di ricreare scenari verosimili nei quali i professionisti operanti nei centri SM (neurologi, infermieri, fisiatri, fisioterapisti, infermieri, psicologi), possano sperimentare in ambiente protetto, gli aspetti principali che possano portare all'apertura e conduzione di un percorso di PCC.

Principali elementi da considerare:

- Quando introdurre o iniziare un percorso di PCC (triggers)
- Come identificare i pazienti che possano essere eleggibili per la PCC (criteri clinici)
- Come condurre il colloquio di PCC con impiego del booklet
- Come garantire che una PCC sia messa in atto (stesura e conservazione del documento di PCC).

Per far emergere gli elementi elencati sopra si sono immaginati 5 scenari clinici da sviluppare, ciascuno dei quali costituirà la "sceneggiatura" di un role playing. In questo documento vengono abbozzate 5 storie tratte da casi clinici veri, che potranno essere modificate, rese più complete o complesse, in base al contributo che i membri del team formativo riterranno di apportare.

15 ottobre 2021

LABORATORIO 1

Setting: visita neurologica di controllo in ambulatorio SM

Protagonisti:

- Paziente con SMSP che si rende conto di avere avuto un peggioramento clinico con risonanza
- magnetica che non evidenzia attività infiammatoria
- Neurologo che deve comunicare al paziente che il peggioramento è in gran parte irreversibile e che
- continuerà nel tempo, e che dimostra l'inefficacia della terapia in atto
  Famigliare che accompagna il paziente, preoccupato per dover fare fronte ad un aumento delle necessità assistenziali
  Infermiere che assiste alla visita

Tematiche:

- il paziente esordisce dicendo al medico: “dottore, mi rendo perfettamente conto che la malattia sta peggiorando, so che non ci sono terapie che possano evitare che io diventi totalmente dipendente. Ho paura di quello che potrebbe succedere e non voglio diventare un vegetale o vivere attaccato a dei tubi” il famigliare interviene minimizzando “non dire così, vedrai che andrà tutto bene, cosa vuoi
- che ti possa dire il dottore? Sai che noi ti vogliamo bene e che troveremo una soluzione a tutto. Il
- paziente: dottore, ma se poi io non riuscissi più a parlare o a comunicare, come faccio ad essere sicuro che non mi venga fatto un accanimento terapeutico?

***Mandato**:* proporre un percorso di PCC su input di un paziente competente che lo richieda.

Svolgimento: il neurologo, con il contributo dell’infermiere, sonda quanto il paziente voglia sapere, propone lo studio ConCure illustrando l’informativa ad esso relativa, rassicura la diade sulla legittimità della PCC e pianifica il follow up.

LABORATORIO 2

Setting: visita neurologica di controllo in ambulatorio SM

Protagonisti:

- Famigliare preoccupato per il peggioramento del paziente, in quel momento impegnato in altra
- attività al centro (valutazione funzionale della disfagia)
- Neurologo
- Psicologo
  Paziente (arriva dopo il colloquio tra familiare neurologo e psicologo, in carrozzina)
  Infermiere (accompagna il paziente)

Tematiche:

- Il famigliare chiede il colloquio perché si rende conto del peggioramento clinico del suo caro, ha paura della disfagia, teme che peggiori e che possa causare soffocamento. Non ha chiaro quanto il paziente si renda conto di questa possibilità, non sa cosa fare nel caso questo capiti, come parlargliene. Ha paura che il peggioramento lo esponga a tentativi anticonservativi, perché in passato aveva detto: “Se non riuscirò più a mangiare, piuttosto che farmi intubare preferisco buttarmi sotto al treno”
- Il neurologo e lo psicologo non hanno ancora informazioni chiare sulla disfagia, sul rischio attuale di aspirazione. Sanno però che il paziente ha una tosse che perde di efficacia, lo pneumologo ha anche ipotizzato l’utilizzo della macchina della tosse, ma al paziente questo non è ancora stato comunicato

Mandato: come gestire una situazione di possibile PCC in una situazione relazionale difficile?

Svolgimento: il neurologo e lo psicologo propongono al famigliare lo studio ConCure. Arriva il paziente accompagnato dall’infermiere e chiede di cosa stessero parlando…

16 ottobre 2021

LABORATORIO 3

Setting: visita di controllo fissata per iniziare il percorso di PCC.

Premessa (a cura del facilitatore): Durante una precedente seduta di riabilitazione fisioterapica e adattamento all'utilizzo di un ausilio per la mobilizzazione, il fisioterapista ha chiesto al paziente se e quanto si sia sentito in difficoltà nella mobilizzazione nell'ultimo periodo. Il paziente ha ammesso che per lui è sempre più difficile muoversi, che necessita di appoggi, che è inciampato in casa un paio di volte rischiando di cadere, ma che ha il terrore di finire in carrozzina e perdere definitivamente l'uso delle gambe. Il terapista ha, a questo punto, proposto degli scenari che consentano di mantenere alcuni gradi di autonomia, anche attraverso ausili. Il paziente ha risposto che tanto è tutto inutile, che la sua volontà non sarà assecondata e che prima o poi finirà inevitabilmente in una struttura per non autosufficienti, perché tanto nessuno si occuperà di lui quando avrà perso l'autonomia. Neanche Dio potrà aiutarlo in questa situazione e questo ha messo in crisi i suoi valori, la sua fede ed il senso della sua vita. In seguito alla condivisione di questi pensieri, il terapista ha proposto di organizzare un incontro con il neurologo di fiducia, per parlargli di una possibile pianificazione condivisa delle cure. Il paziente ha accettato e, nell'incontro inscenato in questo role-playing, sa che bisogna affrontare l'argomento della PCC.

Protagonisti:
- Paziente con SMSP che sta perdendo autonomia nella deambulazione ed è a rischio di frattura
- Fisioterapista del centro SM che ha proposto al paziente di parlare con il suo neurologo di queste
- preoccupazioni
  Neurologo

Mandato: iniziare una discussione di PCC, utilizzando – se possibile – il booklet-ConCure, per affrontare le questioni relative ai valori di riferimento, alla spiritualità e alle credenze del paziente e alle scelte che potrà dover prendere, se lo vorrà.

Svolgimento: attraverso l'accoglienza dei temi emersi il neurologo, insieme ai componenti del team, propone ilbooklet ConCure come strumento di riflessione sugli aspetti valoriali, spirituali del paziente e utilizza la parte di pianificazione soprattutto per quanto riguarda le scelte verso i possibili setting di cura.

LABORATORIO 4

Setting*:* visita fisiatrica di controllo con paziente con SM progressiva, in fase di relativa stabilità fisica, ma con unlivello di compromissione cognitiva e comunicativa in peggioramento.

Premessa (a cura del facilitatore): Durante una riunione di equipe svoltasi una settimana prima, il neuropsicologo ha portato il caso di un paziente che sta facendo delle sedute di supporto. Ha riferito di essere preoccupato, perché ha percepito che il paziente mostra un disorientamento temporale, mentre l'orientamento personale e familiare è apparso adeguato. Lo psicologo ha anche riportato che l'eloquio spontaneo risulta lievemente rallentato, corretto nella forma e nei contenuti, quasi sempre pertinente rispetto al contesto. Ha giudicato estremamente ridotta l'intenzionalità comunicativa, pur permanendo una risposta adeguata a domanda diretta. Il tono dell'umore e il comportamento gli sono apparsi, invece, congrui alla situazione di colloquio e di valutazione. Il neuropsicologo ha riportato anche che il paziente gli ha riferito lievi difficoltà di memoria e, secondo lo psicologo, non sembra pienamente consapevole delle fragilità osservate dall'equipe durante le valutazioni. Il fisiatra ha commentato dicendo che il paziente gli aveva precedentemente detto di voler assolutamente fare 'il testamento biologico, o come si chiama…' e che, quindi, sarebbe il caso di iniziare una PCC L'infermiere ha mostrato i suoi dubbi rispetto alla capacità del paziente di prendere decisioni importanti per sé sull'assistenza e i trattamenti, ma il terapista ha suggerito che bisognerebbe almeno provare, anche se ha dei dubbi se sia in grado di firmare la parte relativa alla PCC inclusa nel booklet. Nell'incontro inscenato in questo role-playing, il fisiatra ha proposto al paziente di incontrarsi per affrontare insieme una discussione relativa all'evoluzione della sua malattia e alla possibilità di prendere insieme alcune decisioni. Il paziente è stato parzialmente introdotto alla questione anche dal neuropsicologo.

Protagonisti

- Fisiatra
- Infermiere
- Neuropsicologo
- Fisioterapista

***Mandato**:* valutare la competenza cognitiva ed emotiva per iniziare una PCC e proporgli l'eventuale utilizzo del booklet. ***Svolgimento**:* durante il colloquio, il medico con l'aiuto dei colleghi, valuta la mental capacity del paziente, facilita la comunicazione con lui, gli propone di identificare un fiduciario e di iniziare una PCC, se lo desidera.

LABORATORIO 5

Setting: visita neurologica in ambulatorio SM per impostare una PCC ***Premessa (a cura del facilitatore):***
Questo scenario descrive il canovaccio di un possibile colloquio di PCC tramite l'utilizzo del booklet.
In un incontro precedente il paziente, resosi conto di un peggioramento evidente, ha mostrato preoccupazioni sul futuro e sulla possibilità di perdere autonomia, fino al punto di dipendere da macchinari o non riuscire più a comunicare le sue volontà. L'incontro odierno è stato, quindi, pianificato per iniziare, insieme, una PCC. Il paziente ha compreso l'obiettivo dell'incontro e ha accettato di iniziare questo percorso, ma non ha mai visto il booklet.

***Partecipanti**:*

- Neurologo
- Infermiere
- Paziente
- Moglie del paziente (presente su suggerimento del neurologo; non era presente durante il colloquio precedente, quindi possiede solo informazioni che le sono state date dal marito).

Mandato: impostare una PCC utilizzando il booklet, allo scopo di raccogliere le firme da conservare in cartella clinica.

Svolgimento:

- il neurologo esordisce ripercorrendo, a grandi linee, i diritti che la L. n.219 del 2017 prevede e le motivazioni per cui ha ritenuto che questo booklet potesse aiutare il paziente (il signor Giovanni). Spiega alla moglie quale potrebbe essere, se lei lo accetta, il suo ruolo in questo percorso di PCC;
- Il paziente conferma la sua volontà di mettere per iscritto le sue preferenze, perché questo gli dà la sensazione di tenere sotto controllo la sua vita. Nonostante questo, non ha mai riflettuto su come le cose per lui importanti si traducano in scelte precise. La moglie accetta di aiutarlo perché hanno sempre affrontato la malattia in due, ma precisa che sui trattamenti alla fine della vita hanno, probabilmente, alcune idee differenti. Tuttavia, è la prima volta che ne parlano in maniera così esplicita.

Step da seguire:

- Panoramica delle sezioni che compongono il booklet; rassicurazione sulla modularità del documento e sulla gradualità del percorso;
- Il neurologo illustra come è organizzato il booklet: la guida a sinistra, che rappresenta degli esempi utili a compilare la parte a destra.
- Ciascuna sezione viene letta insieme e compilata insieme, cercando di seguire l'ordine delle sezioni previste, ma assecondando il paziente se qualcuna gli sembra ridondante o troppo pesante;
- Particolare attenzione alla sezione dalla numero 5 in poi: importanza di soffermarsi sul significato di alcuni termini tecnici in merito ai trattamenti; importanza di spiegare il ruolo del fiduciario;
- La sezione 8 rappresenta la parte del documento che riassume quanto condiviso negli step precedenti e esplicita le decisioni specifiche: è il momento conclusivo del role-play (potrebbero non arrivarci, non importa, chiarire questo aspetto).

ConCure-SM Fase 2

Training Residenzialedegli Operatori
Milano, AmadeoLab

Guida per l'Osservatore (Laboratori di role playing)

Mandato generale: concentrati nell'osservare l'interazione e poni attenzione agli aspetti dello scambio comunicativo tra le parti coinvolte che ti hanno particolarmente colpito.

Mandati specifici:

1. Coinvolgimento del paziente (e familiare se presente):
   - quanto è stato coinvolto il paziente nel processo decisionale
   - come è stata esplorata ed elicitata la sua prospettiva

2. Contenuti teorici:
   - in quali passaggi dello scambio comunicativo hai ritrovato quanto proposto nel corso della mattinata di venerdì

3. Bisogni informativi del paziente (e familiare se presente):
   - quali erano a tuo parere i bisogni informativi del paziente
   - se e come sono stati esplorati ed elicitati

4. Bisogni emotivi del paziente (e familiare se presente):
   - in che modo il paziente ha espresso il suo stato d'animo
   - in quali passaggi hai rilevato una particolare tensione emotiva
   - come è stata gestita

5. Coerenza della comunicazione interprofessionale:
   - quanto il messaggio inviato al paziente dalle diverse figure professionali risulta coerente

6. Ascolto e accoglienza:
   - in che misura il medico/equipe ha trasmesso ascolto attivo e passivo al paziente
   - in quali passaggi hai notato che i bisogni e le aspettative del paziente venivano/non venivano accolti

7. Interventi comunicativi facilitanti e inibenti:
   - cosa ha favorito la comunicazione
   - cosa l'ha ostacolata

8. Comprensione delle informazioni fornite:
   - se e come è stata verificata la comprensione del paziente delle informazioni a lui fornite

9. Restituzione/Chiusura dell'incontro:
   - in che misura l'incontro è stato chiuso in modo adeguato (es. ricapitolazione delle decisioni prese, pianificazione di un incontro successivo, consegna al paziente di recapito telefonico o email cui riferirsi in caso di necessità)
