# Supplementary material for: Communication competences of multiple sclerosis neurologists during advance care planning conversations: A multi-observer study
Source: PLoS One. 2026 Mar 12;21(3):e0336183. doi: 10.1371/journal.pone.0336183 (PMC12981511; doi:10.1371/journal.pone.0336183)
Supplement: S1 Table — (DOCX) [file pone.0336183.s002.docx]

**S1 Table.** The seven sub-categories of cues in the Verona Coding Definitions of Emotional Sequences (VR-CoDES).

| 1. Words or phrases in which the patient uses vague or unspecified words to describe his/her emotions. |
| --- |
| 1. Verbal hints to hidden concerns (emphasizing, unusual words, unusual description of symptoms, profanities, exclamations, metaphors, ambiguous words, double negatives, expressions of uncertainties and hope). |
| 1. Words or phrases which emphasize (verbally or non-verbally) physiological or cognitive correlates (regarding sleep, appetite, physical energy, excitement or motor slowing down, sexual desire, concentration) of unpleasant emotional states. Physiological correlates may be described by words such as weak, dizzy, tense, restless, or by reports of crying whereas cognitive correlates may be described by words such as poor concentration or poor memory. |
| 1. Neutral expressions that mention issues of potential emotional importance which stand out from the narrative background and refer to stressful life events and conditions. This applies to non-verbal emphasis of the sentence, abrupt introduction of new content, pauses before or after the expression, or to a patient-elicited repetition of a previous neutral expression in subsequent turns. |
| 1. A repetition, with very similar words, of an expression said in a previous turn by the patient. |
